# Promoting Adolescents’ Heritage Cultural Identity Development: Exploring the Role of Autonomy and Relatedness Satisfaction in School-Based Interventions

**DOI:** 10.1007/s10964-024-02017-3

**Published:** 2024-05-24

**Authors:** Sophie I. E. Hölscher, Maja K. Schachner, Linda P. Juang, Gianmarco Altoè

**Affiliations:** 1 Martin-Luther-Universität, Halle-Wittenberg, Germany; 2 Universität Potsdam, Potsdam, Germany; 3 University of Padova, Padova, Italy

**Keywords:** Need satisfaction, Intervention, Ethnic-racial identity development, Adolescence, School, Identity Project

## Abstract

Given the significance of heritage cultural identity for optimal adolescent development, it is imperative to investigate factors influencing the efficacy of interventions aimed at promoting heritage cultural identity. Using latent profile cluster analysis and multinomial logistic regressions, this longitudinal study examined how autonomy and relatedness need satisfaction at school (1) related to heritage cultural identity development trajectories, and (2) moderated effects of a school-based intervention. The study included *N* = 198 adolescents (*M*_age_ = 12.86 years, *SD*_age_ = 0.75, 52% female, 41% immigrant descent, 49% intervention group) in Germany. Teacher-student relationships played an important role in facilitating intervention effects on identity development trajectories, emphasizing the importance of the relational context when implementing school-based interventions to promote heritage cultural identity development.

## Introduction

Ethnic-racial or heritage cultural identity^1^ reflects the attitudes and beliefs individuals have about their ethnic, racial or cultural group memberships, as well as the processes by which individuals explore, develop, and maintain this facet of their identity over time (Umaña‐Taylor et al., 2014). The search for (ethnic-racial/heritage cultural) identity is a critical developmental task in adolescence (Erikson, 1968), and developing a stable identity carries significant implications throughout the life span, as it is associated with high-quality relationships, academic achievement, better psychological well-being, and higher life satisfaction (Crocetti, 2017). Recognizing this, school-based interventions have been designed to support adolescents in their ethnic-racial or heritage cultural identity development (e.g., the *Identity Project;* Umaña-Taylor & Douglass, 2017). However, investigating factors influencing the efficacy of such interventions, specifically the context in which it is implemented (Walton & Yeager, 2020), is essential to ensure optimal identity development of all adolescents. The current study focuses on the pivotal role autonomy and relatedness play in the process of identity development, as individuals’ social environment can either foster or hinder the development of specific identity facets (Ryan & Deci, 2017). Therefore, the aim of this study is to investigate how adolescents’ experiences of autonomy and relatedness within the school context facilitate the development of ethnic racial or heritage cultural identity in adolescents, and how it may enhance the efficacy of school-based interventions targeting identity development, such as the Identity Project.

This study focuses on three key aspects of ethnic-racial identity development: *exploration* (i.e., actively questioning potential identity alternatives), *resolution* (i.e., a sense of clarity regarding the personal meaning of their identity), and *affirmation* (i.e., the extent to which individuals feel positively about their identity) (Umaña-Taylor et al., 2004), as they have all persistently demonstrated positive associations with socioemotional adjustment in adolescents (Rivas-Drake et al., 2014). Going beyond research on ethnic-racial and heritage cultural identity that often focuses on average levels of identity scores, the current study seeks to identify trajectories of heritage cultural identity development in adolescents. It specifically examines how the satisfaction of the basic needs for autonomy and relatedness^2^ (Self-Determination Theory; Ryan & Deci, 2017) within the school context may influence the developmental paths of exploration, resolution, and affirmation in heritage cultural identity. Previous research has shown that autonomy and relatedness satisfaction facilitate the development of a (vocational) identity that better represents adolescents’ inner values and wishes (Soenens & Vansteenkiste, 2023), as basic need satisfaction provides them with the energy necessary to engage in identity development related efforts (Luyckx et al., 2009). Furthermore, the approach of Culturally Sustaining Pedagogy, aimed at promoting heritage cultural identity within pedagogical contexts by emphasizing the retention of marginalized students’ heritage culture, highlights student autonomy and relatedness as important features of settings aiming to enact Culturally Sustaining Pedagogy (Paris, 2021). Therefore, this study posits that satisfaction of the basic needs for autonomy, as measured by perceived autonomy support of teachers and intrinsic motivation, and relatedness, as measured by perceived relatedness support of teachers and peer belonging, also promotes ethnic-racial and heritage cultural identity development processes, and may play an important role in promoting the efficacy of school-based interventions such as the Identity Project. By capturing trajectories of heritage cultural identity development, testing for effects on affirmation as an additional facet of heritage cultural identity development not investigated in most intervention studies promoting ethnic-racial or heritage cultural identity (for an exception see Abdullahi et al., 2023), and exploring the potential interaction between need satisfaction in the school context and a school-based intervention aimed at promoting ethnic-racial and heritage cultural identity development, the current study can offer a deeper understanding of the dynamics involved in heritage cultural identity development and provide valuable insights into effective strategies for supporting adolescents on their journey of heritage cultural identity development.

### Ethnic-racial Identity Development in Adolescence

Exploration, resolution and affirmation are important facets of ethnic-racial identity development, as they have been consistently found to be related to better well-being in adolescents. Ethnic-racial identity exploration is essential for adolescents to gain their own understanding of their ethnic and racial background, rather than passively adopting the perspectives of significant others, and has been associated with higher self-esteem and less depressive symptoms (Rivas-Drake et al., 2014). Research further shows that adolescents engage in the most favorable form of ethnic-racial identity development when choosing an identity via meaningful exploration, as that may lead to ethnic-racial identity resolution (Umaña-Taylor et al., 2018). Identity resolution allows individuals to achieve a secure identity in which they experience wholeness and a sense of continuity between their past, current and future self, and has many psychological benefits, such as better well-being and functioning, and less psychological distress (Umaña-Taylor & Douglass, 2017). Ethnic-racial identity affirmation, too, has been consistently related to positive psychological functioning and mental health among adolescents (Rivas-Drake et al., 2014), as positive perceptions of the social group(s) one belongs to plays an important role in upholding a positive self-image (Tajfel & Turner, 1979).

Research has shown that changes in ethnic-racial or heritage cultural identity over time, specifically distinct trajectory patterns, offer a more comprehensive understanding of identity processes than solely focusing on average levels of identity scores. This research often identifies between two and four ethnic-racial (e.g., Chavous et al., 2018) or heritage cultural (e.g., Juang et al., 2024) identity trajectories, with some common patterns including low, medium, and high trajectories. However, these trajectories can differ in terms of direction (e.g., stable, increasing or decreasing), and these differences are influenced by several factors such as the age group being studied, the time intervals between assessments, and external factors such as the school climate (Juang et al., 2024), experiences of discrimination or friendships (Chavous et al., 2018). As the school environment is a significant context for the development of identities (Schachner et al., 2016), it is crucial to pinpoint the factors contributing to ethnic-racial identity development within the school environment. This study focuses on one such condition: the satisfaction of basic psychological needs, which may also serve as a moderating factor in interventions aimed at enhancing ethnic-racial identity development.

### Ethnic-Racial Identity Development in the German Context

In Germany, instead of race and ethnicity, the discourse focuses on categorizing individuals and groups based on culture and descent. Following the racist persecution policies under National Socialism, references to the category of race or ethnicity were considered taboo and increasingly sanctioned in public and political discourse. The concept of culture and immigrant descent offered alternative frames of reference for collectively homogenizing individuals and assigning them specific characteristics and traits, functioning as a substitute for race due to its conceptual vagueness, and operating with similar biological assumptions on the supposedly hereditary nature of culture within groups (DeZim, 2023). In consequence, non-Christian-secular, non-white, individuals are racially minoritized in the German context and categorized as “of immigrant descent”, while the “German” identity is reserved for Christian-secular, white, non-immigrant individuals (Moffitt & Juang, 2019).

Taking into account these socio-cultural variations in the understanding of ethnicity, race, and culture, ERI is conceptualized as heritage cultural identity in the German context. Heritage cultural identity encompasses the thoughts and feelings individuals have about being a part of their heritage cultural groups. It refers to the familial background of a person, and can be based on national (e.g., German), regional (e.g., Bavarian), ethnic (e.g., Sorbian), and/or religious (e.g., Muslim) affiliation(s) most relevant to the self-concept of individuals. “Heritage cultural identity” substantially overlaps with, but is not the same as, the construct of ethnic-racial identity (Juang et al., 2021). In German mainstream society, including schools, openly discussing race-related experiences remains a taboo (Juang et al., 2021). Consequently, the formation of racial identities, as commonly observed in the United States, is less prevalent in Germany. Instead, individuals tend to develop racialized identities based on other significant social categories such as heritage culture, migration status, or religion (Juang et al., 2021). Although the term “heritage cultural identity” does not explicitly acknowledge the racialized experiences in the German context, these identities operate similarly to race in the United States, leading to groups being disadvantaged or privileged based on these racialized identities (Foner, 2015). As a result, experiences related to race remain significant in Germany, as in the United States.

These racialized experiences often manifest within the school context. While the conceptualization and discourse surrounding ethnicity, race, and culture may vary between contexts, the lived experiences and challenges faced by students of immigrant descent in Germany mirror those of racially minoritized adolescents in the United States. Students of immigrant descent experience additional challenges in the school context, such as stereotypes, lower academic expectations of teachers, individual-level racism, and lower well-being in school (Kunyu et al., 2020), as well as structural racism highlighted by the disproportionate lack of access to educational tracks enabling university admission among students of immigrant descent (Reiss et al., 2019). The parallels between ethnic-racial and heritage cultural identity are also seen in the positive well-being outcomes associated with heritage cultural identity. Similar to racially minoritized adolescents in the United States, adolescents of immigrant descent in Germany who have a stronger connection to their heritage identities tend to experience better psychological well-being, as evidenced by fewer depressive symptoms, increased life satisfaction, and higher self-esteem (Juang et al., 2023a).

However, the educational landscape in Germany is marked by assimilative educational strategies, including the promotion of monolingualism within classrooms (Gries et al., 2021), coupled with egalitarian principles that reflect a tendency to avoid discussions of race and maintain superficial forms of multiculturalism (Civitillo et al., 2017). This may impede the ability of all students to equally navigate and express their diverse cultural backgrounds in the school context. Together, these factors sustain systemic discrimination and impede equal opportunities for success and support, as well as heritage cultural identity development among racially minoritized students of immigrant descent. This underscores the importance of implementing school-based interventions that may provide spaces for racially minoritized adolescents to engage with their heritage cultural identity, as well as to investigating factors, such as basic need satisfaction of autonomy and relatedness, that may promote heritage cultural identity development in school-based interventions.

### Basic Need satisfaction and Identity Development

Self-Determination Theory (Ryan & Deci, 2017) and Erikson’s identity framework (1968) emphasize the significance of the basic psychological needs for autonomy and relatedness as a key concept for identity development across various cultures (Vansteenkiste et al., 2020). Autonomy refers to the need to engage in behavior willingly, while relatedness (i.e., belonging) pertains to feeling connected to others. When the need for autonomy is satisfied, individuals feel a sense of psychological freedom, while the satisfaction of relatedness results in individuals feeling supported by a social network (Vansteenkiste et al., 2020). According to Self-Determination Theory (Ryan & Deci, 2017) the basic psychological needs for autonomy and relatedness are important for identity development because exploring, gathering and comparing multiple potential identity options requires a lot of effort, as does the internalization and acceptance of a chosen identity aspect. The energy for these processes may be provided by the satisfaction of the basic needs for autonomy and relatedness. Research specifically emphasizes the role of schools in meeting the needs for autonomy and relatedness in adolescence (stage-environment fit, Eccles & Roeser, 2011). When schools support students’ needs, e.g., prioritize adolescents’ autonomy and choice in their learning and encourage their social connections with peers and teachers, this is more likely to foster positive developmental outcomes in adolescents, such as identity development. The satisfaction of adolescents’ basic psychological needs by their environment is of particular importance, due to the many important transitions and changes that occur during this stage of development (Laporte et al., 2021).

Empirical research underlines the importance of autonomy and relatedness satisfaction as energizing processes within vocational identity development, which drive individuals to proactively explore different identity options followed by choosing certain identities (Luyckx et al., 2009). When individuals can act autonomously, i.e., have the freedom to align their actions with their own interests and values, they are more inclined to explore identity options and evaluate identity-relevant information. When the need for relatedness is satisfied and individuals feel they are being supported by a social network, this likely provides individuals with the necessary feelings of psychological security needed for them to explore their environment (i.e., exploration, Soenens & Vansteenkiste, 2023). Moreover, the satisfaction of relatedness is believed to be crucial for individuals to understand what their chosen identities mean to them (i.e., resolution), and when important others support individuals’ chosen identities, it allows them to become more strongly convinced of their chosen identity (i.e., affirmation, Soenens & Vansteenkiste, 2023). So, feeling autonomous and being connected to and supported by a social network helps individuals in exploring, resolving and affirming their identity.

The present study posits that the relationship between autonomy and relatedness satisfaction and vocational identity development will transfer to heritage cultural identity development processes for adolescents. This assertion is supported by the framework of Culturally Sustaining Pedagogy. Culturally Sustaining Pedagogy prioritizes the centering of heritage cultures, including languages, practices, and knowledge (Paris, 2021). By emphasizing marginalized students’ heritage cultural and linguistic practices, Culturally Sustaining Pedagogy aims to promote heritage cultural identity within pedagogical contexts, including the school environment. This framework underscores the significance of student agency (i.e., autonomy) and positive relationships (i.e., relatedness) as pivotal features of settings where Culturally Sustaining Pedagogy is implemented. Through fostering autonomy and facilitating strong relationships, Culturally Sustaining Pedagogy provides an environment conducive to the exploration and affirmation of heritage cultural identities among adolescents (Paris, 2021).

Autonomy may be satisfied within the school context both by perceived autonomy support of teachers and intrinsic academic motivation. Relatedness within the school context may be satisfied by relationships with teachers (perceived teacher support of relatedness) and peers (peer belonging). If adolescents’ basic psychological needs are satisfied in the school context, this may provide them with the necessary energy and safety needed to invest in identity-related efforts and make them more likely to explore the different heritage cultural identity options at hand (exploration), to have a clearer sense of the personal meaning of their heritage cultural identity (resolution), and feel more positively about their heritage cultural identity (affirmation) over time, resulting in higher or more increasing heritage cultural identity trajectories. As such, the study expects that this need satisfaction will moderate the effect of school-based interventions aimed at promoting ethnic-racial or heritage cultural identity on these trajectories, influencing the way in which interventions contribute to the development of heritage cultural identity in adolescents.

#### Autonomy satisfaction and identity development

Various theoretical and conceptual works underline the importance of autonomous teaching practices and teacher support of autonomy for identity development processes (e.g., Flum & Kaplan, 2012). Namely, children and adolescents encounter a variety of school experiences in which they build their identity by learning about themselves and their interests (La Guardia, 2009). Two separate intervention studies (Faircloth, 2012; Sinai et al., 2012), both designed to trigger global identity exploration (i.e., who one is as a person, across life domains) through educational activities, show that when teachers include content that matters to adolescents and relate it to adolescents’ personal experiences (i.e., autonomous teaching practices), rather than teaching in an over-structured manner focusing only on academic studies (i.e., controlling teaching practices), adolescents engage in more global identity exploration.

Additionally, much research with older adolescents and young adults has investigated how academic intrinsic motivation, a crucial facet of autonomy involving pursuing activities aligned with individuals’ interests, values, and goals, relates to global and vocational identity development. Adolescents with a high level of intrinsic academic motivation are more agentic, and may use positive exploration to verify the fit of different options with their inner identity (La Guardia, 2009). Cross-sectional and longitudinal empirical studies investigating the relationship between adolescents (academic) intrinsic motivation and vocational identity also show that intrinsic motivation encourages adolescents to commit to particular identity options (i.e., resolution), and to identify more strongly with these chosen identities (i.e., affirmation, Luyckx et al., 2017). The importance of student autonomy is also highlighted in Culturally Sustaining Pedagogy as important for students to explore and retain their heritage cultures (Paris, 2021). Therefore, it is expected that higher autonomy satisfaction in the school context, as conceptualized by perceived teacher support of autonomy and intrinsic motivation, will be associated with higher or more increasing heritage cultural identity trajectories, and enhance the effect of school-based interventions, specifically the Identity Project, on these trajectories.

#### Relatedness satisfaction and identity development

As part of their school experiences, adolescents develop aspects of their identity while encountering how they themselves and different aspects of their identity are valued by important others such as parents, teachers or peers (La Guardia, 2009). Teachers’ relationships with adolescents, and their responses to the (ethnic-racial/cultural) groups adolescents belong to or the various roles adolescents experiment with can thus promote or impede basic need satisfaction and, hence, the process of identity development (Soenens & Vansteenkiste, 2023). Support from teachers may fulfill adolescents’ need for relatedness and provide the energy necessary to drive an individual’s identity development. The importance of teacher relatedness is also highlighted in the two intervention studies (Faircloth, 2012; Sinai et al., 2012), in which a safe environment was found to be a requirement to motivate adolescents to engage constructively in both academic learning and global identity exploration. Additionally, the importance of good relationships with students in learning settings is highlighted in Culturally Sustaining Pedagogy as pivotal for students to engage with and sustain their heritage cultures (Paris, 2021). Thus, the current study assumes that adolescents who feel supported by their teachers, fulfilling their need for relatedness, feel safe and have the energy to engage in the heritage cultural identity development process.

Other studies focus on the growing importance of peers on identity development during adolescence, showing that feelings of belonging to peers are related to positive identity development, such as better ability to imagine themselves in the future or a more cohesive self-concept. Only few studies have focused on the link between adolescents’ relationships with their peers and the identity development processes (Rageliené, 2016). The limited research available has found that good peer relationships were related to more exploration, resolution and affirmation (see review by Rageliené, 2016). The current study argues that higher relatedness satisfaction of adolescents in the school context, as conceptualized by perceived teacher support of relatedness and peer belonging, will therefore be associated with higher or more increasing heritage cultural identity exploration, resolution and affirmation trajectories, and promote the effect of school-based interventions on these trajectories, using the Identity Project as an example.

### Basic Need Satisfaction and the Identity Project

The Identity Project is a school-based intervention comprised of eight lesson plans (for details see Umaña-Taylor & Douglass, 2017), that focuses on ethnic-racial or heritage cultural identity exploration in adolescents. U.S. longitudinal studies in mid-adolescence demonstrated that the intervention started a cascading process wherein adolescents explored their ethnic–racial identity, gained a better understanding of their ethnic-racial and global identity (resolution), which in turn was linked to positive well-being outcomes (socio-emotional and academic adjustment, outgroup attitudes) one year later (Umaña-Taylor et al., 2018). As the Identity Project allows adolescents to engage in meaningful exploration of their ethnic-racial identity, it may not only foster a clearer understanding of their identity, but could also contribute to a more positive perception of their ethnic-racial identity (affirmation, see Abdullahi et al., 2023). The results of the Identity Project support findings of previous cross-sectional research that ethnic–racial identity exploration is a key mechanism in promoting positive identity-related and well-being outcomes in adolescents (Umaña-Taylor & Rivas-Drake, 2021).

Since then, the Identity Project has been adapted to contexts outside of the United States (for a detailed review, see Juang et al., 2022) and has been implemented in schools across North America and Europe with encouraging but differing effects. Results show varying effects on heritage cultural identity exploration (e.g., Juang et al., 2020), and not all studies observe cascading effects of exploration on identity resolution (e.g., Ceccon et al., 2023). While most studies investigating the effects of the Identity Project have focused on changes in average levels of identity scores, recent studies (Ceccon et al., 2024) have started to examine the effects of the Identity Project on identity trajectories, shedding light on the intervention’s relation to different patterns in heritage cultural identity development.

As the specific conditions in which interventions are carried out play a crucial role (Walton & Yeager, 2020), efficacy of the Identity Project may vary by both contextual and individual factors. The classroom cultural diversity climate (Schachner et al., 2024), family ethnic socialization (Sladek et al., 2021), and the ability to register, process and respond to stimuli (environmental sensitivity; Ceccon et al., 2023) play a significant role in shaping adolescents’ experiences in the Identity Project. Building on the theoretical frameworks highlighting the importance of basic need satisfaction in the school context (Eccles & Roeser, 2011) and empirical support demonstrating the moderating role of autonomy and relatedness in interventions on identity development processes (e.g., Faircloth, 2012), the current study explores how adolescents’ experiences of autonomy (i.e., perceived teacher support of autonomy, intrinsic motivation) and relatedness (i.e., perceived teacher support of relatedness, peer belonging) interacts with the effect of the Identity Project on heritage cultural identity trajectories of adolescents.

As immigrant descent has been highlighted as an important variable that may shape adolescents’ experiences in the Identity Project (e.g., Ceccon et al., 2023), the study will take into account direct and moderating effects of immigrant descent when exploring how adolescents’ experiences of autonomy and relatedness are associated to and interact with the intervention’s effect on heritage cultural identity trajectories. This is particularly pertinent as ethnic-racial or heritage cultural identity plays a more pivotal role for ethnic-racial minoritized adolescents, as they navigate acculturation processes and develop their identity while being confronted with negative stereotypes about their group as well as discrimination (Schachner et al., 2018).

## Current Study

Considering the success of interventions aimed at promoting ethnic-racial and heritage cultural identity development relies not only on the quality of the intervention itself but also on the context in which it is implemented, it is crucial to understand the conditions that optimize the efficacy of school-based interventions designed to foster identity development. If adolescents’ basic psychological needs for autonomy and relatedness are satisfied in the school context, this may provide them with the necessary energy and safety needed to invest in identity-related efforts and sets up the potential for differential trajectories of heritage cultural identity development. Therefore, the current study aims to investigate how adolescents’ levels of basic psychological need satisfaction in the school context influence the trajectories of their heritage cultural identity development during participation in a school-based intervention, the Identity Project. Between two and four distinct heritage cultural identity exploration, resolution and affirmation trajectories for adolescents across the three time points are expected to be identified (research question 1). Next, increased autonomy satisfaction, as indicated by perceived teacher support of autonomy and intrinsic motivation, and increased relatedness satisfaction, as indicated by perceived teacher support of relatedness and belongingness to peers, will predict higher or more increasing heritage cultural identity exploration, resolution and affirmation trajectories (research question 2). Further, it is expected that basic need satisfaction will moderate the effect of the Identity Project intervention, resulting in higher or more increasing heritage cultural identity trajectories when the Identity Project is combined with basic need satisfaction (research question 3). Recognizing the increased salience of heritage cultural identity for adolescents of immigrant descent and given evidence that immigrant descent may have direct implications for identity development as well as moderate effects of the Identity Project on identity development, differences across immigrant descent will be investigated in research questions 2 and 3.

## Methods

### Participants and Procedure

Participants were 386 7^th^ graders (*M*_age_ = 13.04 years, *SD*_age_ = 0.85, 46% female, 48% of immigrant descent, *M*_SES_ = 6.37, *SD*_SES_ = 1.90) from five high schools in Halle (Saale) (*n* = 360) and one high school in Berlin (*n* = 26) in the school year 2021/22. 7^th^ graders were approached as this school year marks the onset of an important transitionary period for students in the federal state of Berlin (see Juang et al., 2020 for more detail). Employing a randomized controlled trial design at the classroom level, the study allocated nine classrooms to the intervention group, where they received the 8-week Identity Project intervention. A questionnaire was distributed in German to both groups one week before the Identity Project was implemented in the intervention group (pretest – T1), one week after the end of the intervention (post-test – T2), and seven weeks after the end of the intervention (follow-up – T3). The ten classrooms in the control group were offered to receive the intervention after the follow-up (T3); however, due to scheduling constraints on the schools’ side, only four control classes opted to partake in the intervention.

Ethical approval was granted by the Berlin Education Senate and the State Board of Education (Landesschulamt) of Saxony-Anhalt. The study targeted schools with a notable percentage of non-German first language adolescents for potential participation. If schools agreed to participate, the Identity Project was presented to 7^th^ grade parents at parent evenings, the informed consent form was shared, and any questions answered. As the Identity Project was taught as part of the regular curriculum, all adolescents in participating classes took part in the project; however, adolescents whose parents did not consent for the accompanying research study did not participate in the survey. Surveys were filled in during 90 min slots within regular class time. Adolescents were given small gifts (e.g., erasers or chocolate) for compensation.

### Measures

Adolescents were asked about basic demographics including their gender, age, cultural self-identification, birthplace, and parents’ birthplace. In line with previous studies (e.g., Schachner et al., 2016), adolescents who were born in Germany from German-born parents were coded as adolescents of non-immigrant descent, and adolescents who were born abroad or in Germany with at least one parent born abroad, were coded as adolescents of immigrant descent. Socioeconomic status was assessed using the family affluence scale (FAS II, see Currie et al., 2008; four items, e.g., “Does your family have a car?”). The scores of individual items were summed to generate a total score, ranging from 0 (indicating the lowest affluence) to 9 (indicating the highest affluence). The main variables are described below, with response scales ranging from 1 = *No, that is not true* to 4 = *Yes, that is true*.

#### Satisfaction of the need for autonomy and relatedness

Autonomy and relatedness satisfaction were measured with two scales each. Perceived autonomy and relatedness support by teachers were assessed using the Teacher as Social Context Questionnaire (Belmont et al., 1992), which was translated into German by the principal investigators and reviewed by a committee of bilingual experts to ensure the accuracy of the translation. The six items with the highest factor loadings from the validation study were selected for each respective subscale. Additionally, autonomy was assessed by an established German scale measuring intrinsic learning motivation (five items, Müller et al., 2007) and relatedness was measured by a peer belonging scale (three items, Skinner et al., 2009), which had been previously used in the German context (e.g., Juang et al., 2020). Example items are “In my class my teacher listens to my ideas” (perceived autonomy support by teachers, ω_T1_ = 0.52, ω_T2_ = 0.63, ω_T3_ = 0.68), “I can count on my teacher when I need him or her” (perceived relatedness support by teachers, ω_T1_ = 0.77, ω_T2_ = 0.78, ω_T3_ = 0.78), “I work and study in the classroom, because I enjoy it” (intrinsic motivation, ω_T1_ = 0.90, ω_T2_ = 0.89, ω_T3_ = 0.89), and “When I am with my classmates, I feel accepted” (peer belonging, ω_T1_ = 0.76, ω_T2_ = 0.83, ω_T3_ = 0.82).

#### Heritage cultural identity

The measurement of heritage cultural identity, defined for participants as the cultural background of an individual’s family, included three items each for exploration and resolution (Ethnic Identity Scale - Brief, Douglass & Umaña Taylor, 2015), and five items for heritage cultural identity affirmation (Leszczensky & Gräbs Santiago, 2015). To fit the scales of exploration and resolution to the German context, mentions of ethnicity were changed to mentions of heritage culture. These scales have been employed in previous research within Germany (e.g., Juang et al., 2020). Example items are “I have attended events that have helped me learn more about my heritage cultural group” (exploration, ω_T1_ = 0.82, ω_T2_ = 0.89, ω_T3_ = 0.89), “I know what my heritage culture means to me” (resolution, ω_T1_ = 0.87, ω_T2_ = 0.87, ω_T3_ = 0.91), and “I am happy about being part of my heritage cultural group” (affirmation, ω_T1_ = 0.89, ω_T2_ = 0.92, ω_T3_ = 0.93).

### Analytic Procedure

As reliable a priori estimates for all effect sizes associated with the research questions were not available, an exploratory, rather than a confirmatory, approach was used. All statistical analyses were conducted using the *R* statistical software (R Core Team, 2022). Pre-registered analyses, data, code of main analyses and supplemental materials are available at the Open Science Framework (https://osf.io/jhcv7/).

As data was collected during the COVID-19 pandemic, there are missing waves of data both on classroom and on individual level. Given the analysis employed to determine longitudinal latent clusters, traditional Full Information Maximum Likelihood (FIML) techniques for handling missing data were not applicable (Fraley et al., 2012). Subsequently, utilizing list-wise deletion, participants who did not complete the control variables (age, gender, immigrant descent, SES), or who did not have data on at least one item value for every main study variable at all three time points were excluded from analyses. In a preliminary analysis, a logistic regression with the presence of missing data as the dependent variable (0 = adolescents who did not have data on at least one item value for every main study variable at one or more time points, 1 = adolescents who completed all main study variables), and gender, age, SES, immigrant descent and the intervention group as independent variables was conducted.

In the main analyses, study variables were considered at all three time points to explore the dynamic interaction of basic need satisfaction and heritage cultural identity throughout the Identity Project. However, to avoid multicollinearity issues, in a first step factor analyses were performed for each of the following variables measured at the three time points: perceived autonomy support by teachers, intrinsic motivation, perceived relatedness support by teachers, and peer belonging. As one clear factor emerged for all variables in question, factor scores were extracted and included in the following analyses as independent variables instead of all three time points per autonomy/relatedness variable. This approach reduced the complexity of the model while still allowing us to account for basic need satisfaction at all three time points. This decision was rooted in the rationale that basic need satisfaction, both before and during the Identity Project, would be associated with heritage cultural identity trajectories. As the intervention was controlled for in the model, this helps ensure that potential confounding effects between basic need satisfaction and the intervention were considered.

In a next step, latent profile cluster analyses (R *mclust* package) were conducted to identify longitudinal trajectories of subjects on the dependent variables (i.e., heritage cultural identity exploration, resolution, and affirmation) across T1, T2 and T3. Latent profile cluster analysis was used to identify possibly meaningful sub-grouping of subjects to help better understand sample heterogeneity (Sterba, 2013). For each identity variable, the presence of one to five trajectories for each identity variable were tested. The inclusion of one cluster in the analysis acted as a baseline model assuming no distinct clusters in the data, and served as a reference point for assessing whether additional clusters significantly enhanced the model fit. On the other end of the spectrum, testing for five clusters ensured that the incorporation of more clusters did not lead to a superior model fit than the commonly found two to four clusters, while at the same time preventing potential data overfitting by testing for more than five clusters. The most plausible number of trajectories was selected based on the Bayesian Information Criteria (BIC; Raftery, 1995).

To investigate whether satisfaction of autonomy and relatedness predicts trajectory membership on each of the three dependent variables, multinomial logistic regressions were run (R *nnet* package). Specifically, for each dependent variable a baseline multinomial logistic regression with the intervention condition, immigrant descent, and the factor scores measuring autonomy (perceived autonomy support by teachers & intrinsic motivation) and relatedness satisfaction (perceived relatedness support by teachers & peer belonging) as independent variables were run. All two-way interactions of autonomy and relatedness satisfaction with the main effect of intervention condition were considered, as well as three-way interactions of autonomy and relatedness satisfaction with intervention condition and immigrant descent. Furthermore, the model included the main effects of gender, age and socioeconomic status as covariates (as is common practice in ethnic-racial identity development studies, e.g., Umaña-Taylor et al., 2018).

Starting from each baseline model (i.e., one for each dependent variable), the best model was selected from observed data using a model selection approach via the R function *step* (R *stats* package) based on the AIC index (Sakamoto et al., 1986). Each best fitting model was evaluated using analysis of deviance (R *car* package) and Odds ratio (Cohen, 1988). Finally, significant interaction effects were interpreted through graphical representations of the trajectory membership probabilities estimated by the model. Detailed information regarding the selected trajectories and multinomial logistic regression models (i.e., descriptives, model parameters, Odds ratio, graphical representations) are available in the supplementary material.

#### Sensitivity analyses

At the multivariate level, to evaluate the presence of influential cases (i.e., cases that have a substantial impact on the estimated model parameters and, consequently, on the interpretation of results), a sensitivity analysis was carried out for each best-fitting multinomial regression model (Hashimoto et al., 2020). For each multinomial regression model and for each case (i.e., observation) included in the model, the difference in log-likelihood between the full model and the model without the case was computed. Cases with a large absolute difference were identified as potential influential cases, and their impact on the model was assessed. If influential cases were found to have a large impact on the model, they were removed, and the differences reported.

## Results

### Preliminary Analyses

A total of *n* = 188 participants had to be excluded due to missing data. Among them, *n* = 35 participants were from two classes that dropped out of the study. Additionally, *n* = 141 participants were excluded due to missing data on main variables, while *n* = 12 participants were removed from analysis due to missing demographic information. The final sample included *N* = 198 adolescents (52% female, 41% of immigrant descent, 49% intervention group, 93% from Halle). A significant difference between the adolescents with and without missing data emerged only in terms of age (*X*^2^(1, *N* = 343) = 11.64, *p* < 0.001). Yet, this difference was relatively small in terms of magnitude, with adolescents in the missing data group having a mean age of 13.25 (*SD* = 0.93), while the final sample without missing data had a mean age of 12.85 (*SD* = 0.75). Nonetheless, this selection bias should be taken into account when interpreting results.

### Heritage Cultural Identity Trajectories

**Fig. 1:**
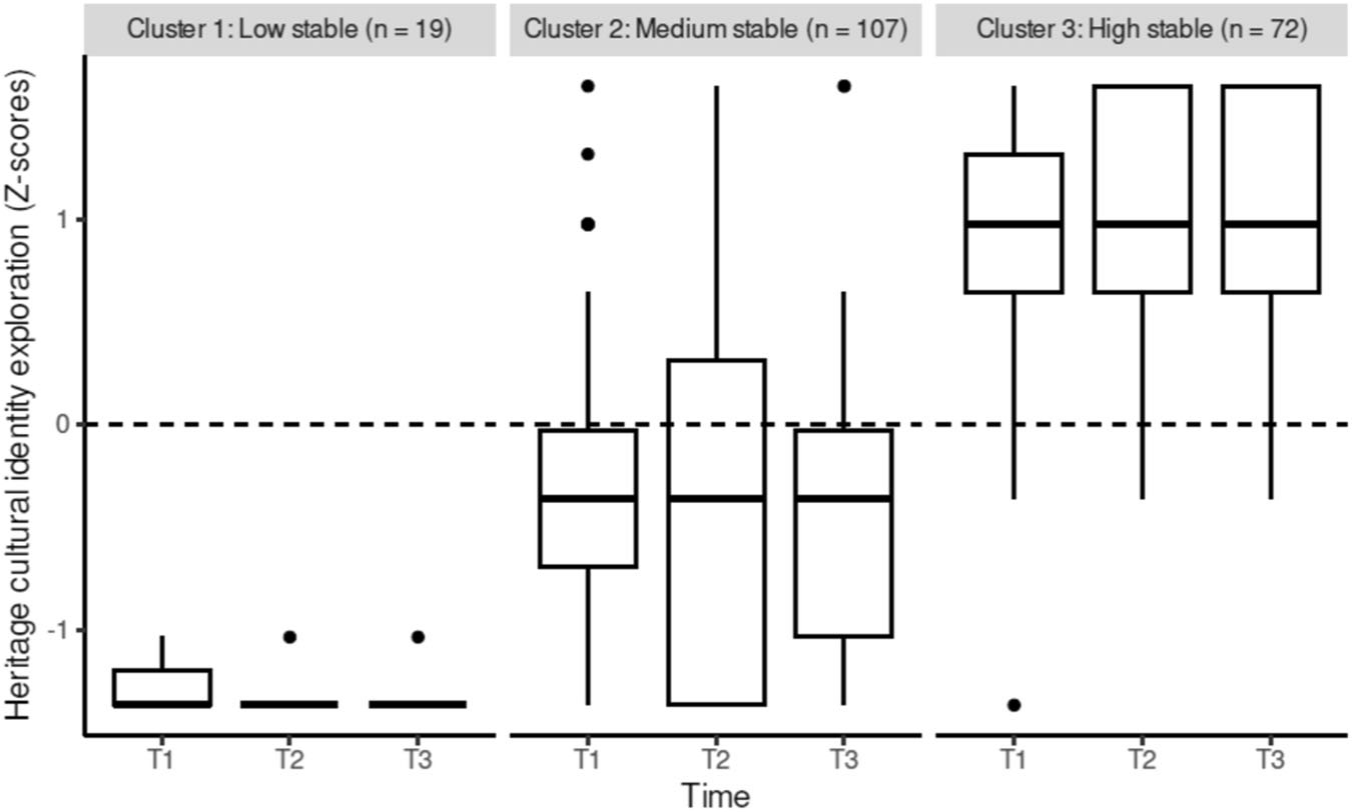
Heritage cultural identity exploration trajectories

**Fig. 2:**
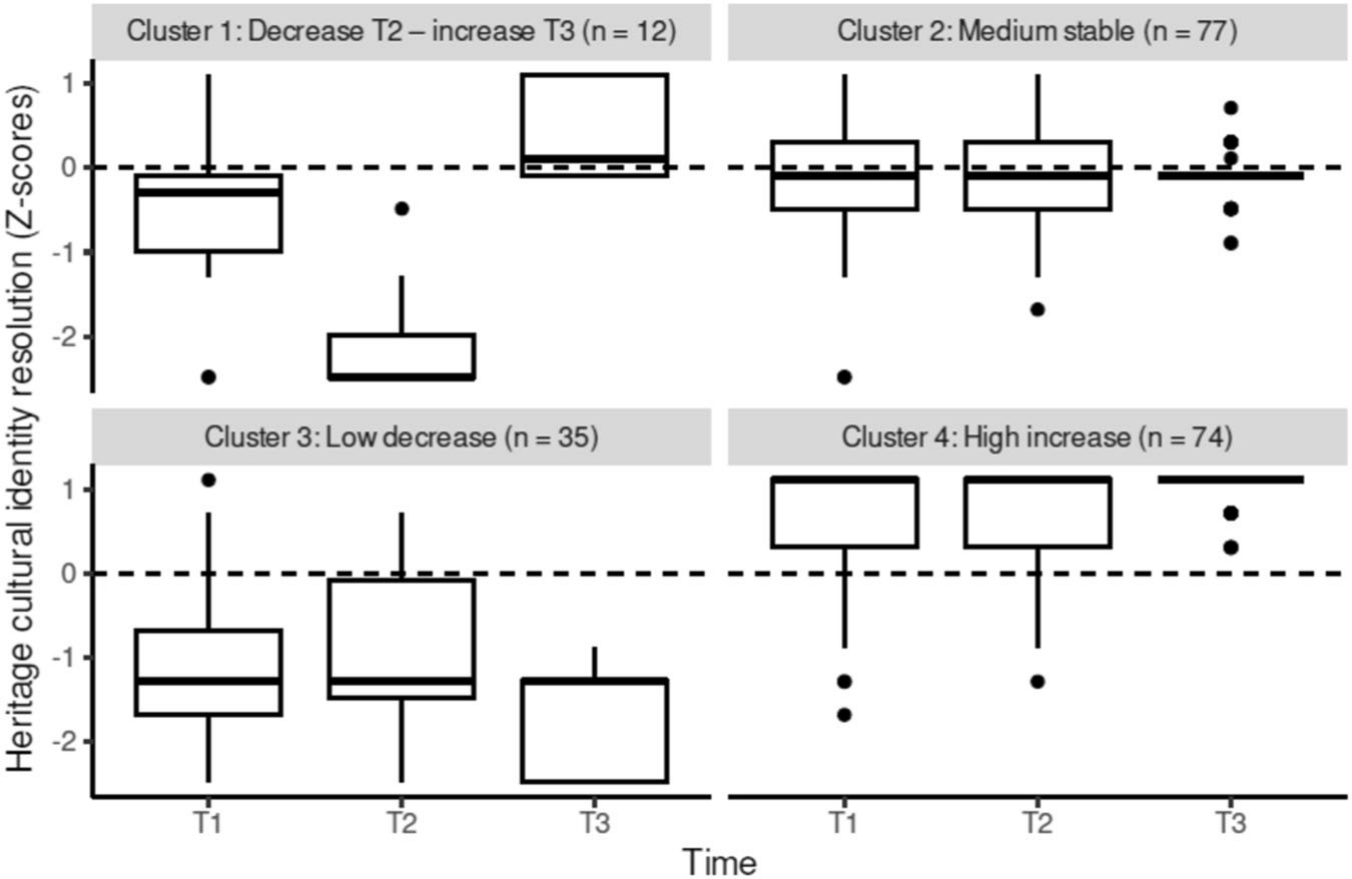
Heritage cultural identity resolution trajectories

**Fig. 3:**
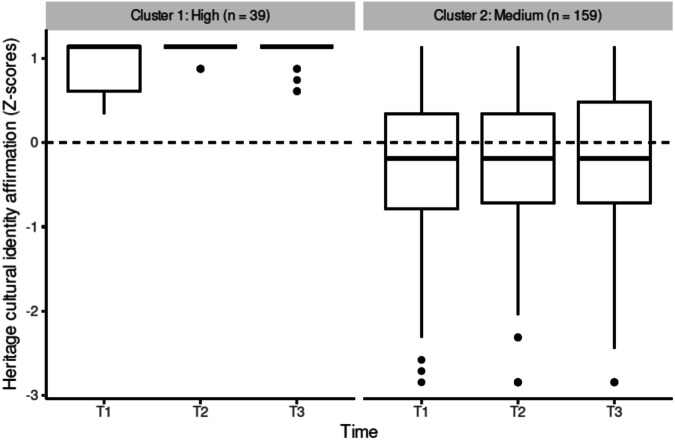
Heritage cultural identity affirmation trajectories

In line with our expectations, between two and four differing heritage cultural identity exploration, resolution and affirmation trajectories for adolescents emerged across the three time points (research question 1). The best solution for heritage cultural identity exploration showed three trajectories (Fig. 1): a low, stable heritage cultural identity exploration trajectory (*n* = 19 adolescents), a medium, stable heritage cultural identity exploration trajectory (*n* = 107 adolescents), and a high, stable heritage cultural identity exploration trajectory (*n* = 72 adolescents). The best solution for heritage cultural identity resolution showed four trajectories (Fig. 2): a trajectory of decrease of heritage cultural identity resolution from T1 to T2, followed by a high increase in heritage cultural identity resolution at T3 (*n* = 12 adolescents), a medium, stable heritage cultural identity resolution trajectory (*n* = 77 adolescents), a low heritage cultural identity resolution trajectory, which decreases further at T3 (*n* = 35 adolescents), and a high heritage cultural identity resolution trajectory, which increases further at T3 (*n* = 74 adolescents). The best solution for heritage cultural identity affirmation showed two trajectories (Fig. 3): a medium, stable heritage cultural identity affirmation trajectory (*n* = 159 adolescents) and a high heritage cultural identity affirmation trajectory, with a slight increase from T1 to T2 (*n* = 39 adolescents).

#### Predicting heritage cultural identity exploration trajectories

The best-fitting multinomial logistic regression model to describe heritage cultural identity exploration trajectories included gender, immigrant descent, intervention, perceived autonomy support by teachers, intrinsic motivation, perceived relatedness support by teachers, peer belonging, and the interactions between intervention and perceived autonomy support by teachers, intervention and perceived relatedness support by teachers, and intervention and peer belonging (*R*^*2*^_*Nagelerke*_ = 0.57). Of these, immigrant descent (*X*^2^(2, *N* = 198) = 30.10, *p* < 0.001), intrinsic motivation (*X*^2^(2, *N* = 198) = 12.04, *p* = 0.002), and the interaction between intervention condition and perceived autonomy support by teachers (*X*^2^(2, *N* = 198) = 8.94, *p* = 0.011), perceived relatedness support by teachers (*X*^2^(2, *N* = 198) = 11.51, *p* = 0.003) and peer belonging (*X*^2^(2, *N* = 198) = 9.11, *p* = 0.011), significantly predicted the heritage cultural identity exploration trajectories. The medium stable heritage culture exploration trajectory was used as a reference category.

There was a main effect of immigrant descent, with adolescents of immigrant descent more likely to be in the high stable and low stable exploration trajectory than adolescents of non-immigrant descent. Also, as expected, when adolescents experience autonomy in the school context, this was associated with higher heritage cultural identity exploration trajectories (research question 2). The higher adolescents’ intrinsic motivation, the less likely they are to be in the low-stable exploration trajectory.

Adolescents’ experiences of autonomy and relatedness across the three time points also moderated the effect of the intervention condition of the Identity Project on heritage cultural exploration trajectories (research question 3). Under conditions of high perceived autonomy support from teachers, control and intervention condition showed opposite effects. In the control condition, the more adolescents perceived teachers to support their autonomy, the less likely adolescents were to be in the low-stable exploration trajectory; but surprisingly, in the intervention condition, the more adolescents perceived teachers to support their autonomy, the *more* likely they were to be in the low-stable exploration trajectory. Under conditions of high perceived relatedness support from teachers, control and intervention condition also showed opposite effects, however, contrary to the interaction results in regards to autonomy support, for the intervention condition the results were in line with expectations. In the intervention condition, the more relatedness support by teachers adolescents reported, the more likely they were to be in the high-stable exploration trajectory, and the higher their feelings of belonging to their peers, the less likely adolescents were to be in the low-stable exploration trajectory. However, in the control condition, the more relatedness support by teachers adolescents’ reported, the less likely adolescents were to be in the high-stable exploration trajectory, and the higher their feelings of belonging to their peers, the more likely adolescents were to be in the low-stable exploration trajectory. The interaction between basic need satisfaction and intervention condition did not differ by immigrant descent of adolescents in regards to the exploration trajectories.

#### Predicting heritage cultural identity resolution trajectories

The best-fitting multinomial logistic regression model identified to describe heritage cultural identity resolution trajectories and test research questions 2 and 3, included immigrant descent, intervention, perceived relatedness support by teachers, peer belonging, and the interactions between intervention and immigrant descent, and intervention and perceived relatedness support by teachers (*R*^*2*^_*Nagelerke*_ = 0.38). Of these, the interaction between the intervention group and immigrant descent (*X*^2^(3, *N* = 198) = 9.50, *p* = 0.023), and the interaction between the intervention group and perceived relatedness support by teachers (*X*^2^(3, *N* = 198) = 7.98, *p* = 0.046) significantly predicted heritage cultural identity resolution trajectories. The medium stable heritage culture resolution trajectory was used as a reference category.

There was no main effect of immigrant descent, nor of autonomy or relatedness satisfaction in the school context on heritage cultural resolution trajectories (research question 2). However, adolescents’ immigrant descent and experiences of relatedness across the three time points moderated the effect of the intervention condition of the Identity Project on heritage cultural resolution trajectories (research question 3). Across conditions, adolescents of immigrant descent were less likely to be in the low-decrease resolution trajectory and more likely to be in the high-increase resolution trajectory than adolescents of non-immigrant descent. This was more pronounced in the control condition, where there are no adolescents of immigrant descent in the low-decrease resolution trajectory, and the highest probability of adolescents of immigrant descent being in the high-increase resolution trajectory. In contrast, adolescents of non-immigrant descent were more likely to be in the high-increase resolution trajectory and less likely to be in the low-decrease resolution trajectory when in the intervention group compared to the control group. Moreover, only under conditions of high perceived relatedness support of teachers, were adolescents in the intervention condition more likely to be in the high increase resolution trajectory. The interaction between basic need satisfaction and intervention condition did not differ by immigrant descent of adolescents in regards to the resolution trajectories.

#### Predicting heritage cultural identity affirmation trajectories

A multinomial logistic regression including all three-way interaction terms (immigrant descent x intervention x relatedness/autonomy satisfaction) did not converge, likely due to the excessive number of parameters to be estimated compared to the structure of the observed data. Further analyses were run comparing logistic regressions including one three-way-interaction each. However, a model without any three-way-interaction was determined to fit the data best (*R*^*2*^_*Nagelerke*_ = 0.57). The best-fitting model to describe heritage cultural identity affirmation trajectories and explore research questions 2 and 3, included age, immigrant descent, intervention, perceived relatedness support by teachers, peer belonging, and an interaction between intervention and perceived relatedness support by teachers. Of these, immigrant descent (*X*^2^(1, *N* = 198) = 50.83, *p* < 0.001), peer belonging (*X*^2^(1, *N* = 198) = 4.26, *p* = 0.039), and the interaction between the intervention group and perceived relatedness support by teachers (*X*^2^(1, *N* = 198) = 6.14, *p* = 0.013) significantly predicted heritage cultural identity affirmation trajectories. The medium heritage culture affirmation trajectory was used as a reference category.

There was a main effect of immigrant descent, with adolescents of immigrant descent more likely to be in the high affirmation trajectory than adolescents of non-immigrant descent. Also, as expected, when adolescents experience relatedness in the school context, this was associated with higher heritage culture affirmation trajectories (research question 2). The higher adolescents’ feelings of belongingness to peers, the more likely they were to be in the high affirmation trajectory.

Adolescents’ experiences of relatedness across the three time points also moderated the effect of the intervention condition of the Identity Project on heritage cultural identity affirmation trajectories (research question 3). Only under conditions of high perceived relatedness support of teachers, were adolescents in the intervention condition more likely to be in the high affirmation trajectory. The interaction between basic need satisfaction and intervention condition did not differ by immigrant descent of adolescents in regards to the affirmation trajectories.

#### Sensitivity analyses

Considering the overall minimal differences between the findings of the best fitting models and the associated models without influential cases, and following a conservative approach, no cases were excluded (for detailed results see Supplementary Material).

## Discussion

School-based interventions offer promising opportunities to promote ethnic-racial and heritage cultural identity development in adolescents, an important developmental task in adolescence (Umaña-Taylor et al., 2018). However, as intervention outcomes hinge not only on the intervention’s quality but also on the implementation context, it becomes crucial to investigate the conditions that optimize the efficacy of school-based interventions in promoting adolescents’ heritage cultural identity development (Walton & Yeager, 2020). Therefore, the current study investigated the importance of basic need satisfaction of autonomy and relatedness in the school context, as highlighted by Self-Determination Theory (Ryan & Deci, 2017), Erikson’s identity framework (1968) and stage-environment fit theory (Eccles & Roeser, 2011), for (1) promoting adolescents’ heritage cultural identity development, and (2) enhancing the effects of a school-based intervention aimed at promoting heritage cultural identity, as exemplified by the Identity Project (Umaña-Taylor & Douglass, 2017). Basic need satisfaction of autonomy and relatedness, particularly teacher-student relationships, emerged as an important condition for school-based interventions to promote positive heritage cultural identity development in adolescents.

The findings support the assumption of differential (between two and four) pathways of heritage cultural identity exploration, resolution and affirmation trajectories emerging for 7^th^ grade adolescents in Germany (research question 1), and shed light on the dynamic and differing development of heritage cultural identity processes in adolescents. They further underline the importance of complementing analyses using average levels of identity scores with analyses investigating identity trajectories. Trajectories go beyond observing how heritage cultural identity changes on average for all adolescents over separate time points, instead revealing how identity develops across these three time points, and how this may differ for various subgroups of adolescents.

In regards to exploration, a substantial number of adolescents exhibited moderate or high stable exploration trajectories, while a smaller group maintained consistently low exploration levels over time. The stable trajectories of exploration are in line with other studies investigating adolescents’ identity trajectories in school-based interventions aimed at promoting heritage cultural identity development (Ceccon et al., 2024) and suggest that how much adolescents actively engage in activities to explore their heritage cultural identity (exploration), may need more time to change than the 16 weeks followed by the current study. As our sample includes younger adolescents, it may also take a longer period of time before there are greater changes in their heritage cultural identity compared to older adolescents (Schachner et al., 2024); the intervention may have initiated exploration and affirmation processes that continue to evolve beyond the 16-week timeframe examined here (Sladek et al., 2021). A lack of change in exploration may also be due to data collection taking place during the COVID-19 pandemic; exploration was measured by activities such as taking part in events to explore your heritage cultural identity, which may have simply not been possible at the time (see also Ceccon et al., 2023).

The more dynamic patterns displayed by trajectories of heritage cultural identity resolution, characterized by both change and stability, also align with recent research investigating adolescents’ identity trajectories in school-based interventions aimed at promoting heritage cultural identity development conducted in Italy (Ceccon et al., 2014) and Sweden (Abdullahi et al., 2023), as well as previous studies examining trajectories of ethnic-racial identity content among Black college students in the United States (Chavous et al., 2018). Though a majority of adolescents followed trajectories characterized by stability or increase, a noteworthy subgroup experienced a temporary dip in resolution post-test, succeeded by a substantial increase at follow-up. The findings show important changes in clarity regarding the personal meaning of heritage cultural identity to adolescents (resolution) over the 16 weeks, suggesting that the process of attaining clarity in one’s cultural identity and its associated meaning is a deeply personal and variable journey, and may be more influenced by individual differences than other identity development processes. The findings further support research suggesting that exploration may not necessarily precede resolution, but resolution may also precede or develop alongside exploration (Crocetti, 2017).

In terms of heritage cultural identity affirmation, findings pointed to two trajectories—a larger group demonstrating a medium and stable affirmation pattern and a smaller group exhibiting high affirmation with a slight increase from the initial to the second assessment point. The stable trajectories of affirmation align with previous research (e.g., Spiegler et al., 2018), that found similar patterns among early adolescents. Stability is the most prevalent pattern of ethnic-racial or heritage cultural identity affirmation during early adolescence (Spiegler et al., 2018), with potential for greater fluctuation in later adolescence (Matsunaga et al., 2010).

Moreover, the findings indicate that these differential pathways emerging for adolescents may vary based on adolescents’ experiences of autonomy and relatedness, as well as their immigrant descent. As expected, the intervention alone does not predict adolescents’ heritage cultural identity trajectories^3^, but adolescents’ heritage cultural identity exploration, resolution and affirmation may vary depending on their immigrant descent and whether they experience autonomy and relatedness in the school context (research question 2). Additionally, participants’ immigrant descent and whether they experience autonomy and relatedness in the school context may shape how school-based interventions relate to heritage cultural exploration, resolution, and affirmation (research question 3). Overall, while the direct effects of autonomy and relatedness on heritage cultural identity development vary in the absence of structured incentives to engage with heritage culture, such as those provided by the Identity Project intervention, the study sheds light on the critical role of teacher-student relationships. These relationships are a fundamental requirement for interventions to effectively foster heritage cultural identity development.

### Basic Need Satisfaction and Heritage Cultural Identity Trajectories

In line with the principles of Culturally Sustaining Pedagogy, which emphasize the importance of student autonomy and positive relationships in retaining and promoting heritage cultural identities within educational settings (Paris, 2021), experiences of autonomy and relatedness across the three time points play an important role in shaping adolescents’ heritage cultural identity trajectories (research question 2). However, contrary to theory (e.g., Ryan & Deci, 2017) and previous studies (e.g., Luyckx et al., 2017) that suggested autonomy’s and relatedness’ role in all three aspects of heritage cultural identity development (exploration, resolution and affirmation), autonomy was only associated with exploration trajectories, and relatedness was only associated with affirmation trajectories. Specifically, in line with expectations, adolescents who experienced more intrinsic motivation were more likely to be in the high heritage cultural identity exploration trajectory. Similarly, the more peer belonging adolescents reported across the three measured time points, the more likely they were to be in the high affirmation trajectory.

These findings underscore the importance of distinguishing between different identity development processes. They suggest that autonomy may play a more significant role in exploration rather than resolution and affirmation, corroborating the assertions of Self-Determination Theory (Ryan & Deci, 2017) that autonomy is crucial for initiating and propelling adolescents’ exploration of their identity. Adolescents whose need for autonomy is fulfilled by intrinsic academic motivation may experience a sense of agency and freedom that could provide them with the necessary energy to invest in identity-related efforts and explore their heritage cultural identity (Luyckx et al., 2009). Forming meaningful connections with peers on the other hand may be more important for affirming adolescents’ heritage cultural identity. Adolescents’ sense of belonging and acceptance within their peer group may foster a sense of belonging (Vansteenkiste & Soenens, 2023) and contribute to a more positive self-perception of their heritage cultural identity (Rageliené, 2016), rather than contributing towards actively exploring or understanding the meaning of their heritage cultural identity. This suggests that experiences of autonomy or relatedness alone may be insufficient for young adolescents to resolve their heritage cultural identity.

Furthermore, the findings emphasize the importance of differentiating between specific types of autonomy and relatedness satisfaction – while intrinsic motivation played a significant role in increasing heritage cultural identity exploration among adolescents, perceived teacher support of autonomy did not. In the same line, while good relationships with peers lead adolescents to feel more positively about their identity, good relationships with their teachers did not. It is possible that in other contexts teacher support of autonomy and relatedness may help adolescents engage in global identity exploration (e.g., Sinai et al., 2012); however, in German school contexts that are characterized by an assimilationist climate and lack explicit emphases on heritage cultural identity (Gries et al., 2021), this support may not be sufficient for adolescents to actively explore their heritage cultural identity or develop positive attitudes towards their heritage cultural identity. This may be different in other aspects of identity, such as vocational identity, which receives ample emphasis and encouragement within educational contexts, such as career counseling or vocational training programs. However, school interventions such as the Identity Project offer a promising solution to this limitation by providing the necessary incentives and support for adolescents to actively engage with their heritage cultural identity within the school environment.

Additionally, adolescents of immigrant descent exhibited unique trajectories, indicating the influence of acculturation processes, negative stereotypes, and discrimination on their heritage cultural identity development. Adolescents of immigrant descent were more likely to be in the high exploration and affirmation trajectories than adolescents of non-immigrant descent. Yet, they were also more at risk of being in the low exploration trajectory, although this was the least common of the exploration trajectories. This follows the pattern of previous research showing that ethnic-racial identity is more salient for minoritized adolescents, as they develop their heritage cultural identity while navigating additional challenges such as discrimination (Schachner et al., 2018). Adolescents’ response to these experiences of discrimination and social rejection may vary based on the strategies available to them. Encountering discrimination can signal to adolescents that they do not belong to and are not welcome in the national identity. To protect their well-being and self-esteem, adolescents may therefore dis-identify with the national identity and identify more strongly with their heritage cultural group, leading to higher heritage cultural identity exploration and affirmation (Tajfel & Turner, 1979).

On the other hand, in response to social rejection adolescents may identify strongly with the larger national identity while distancing themselves from their heritage cultural identity (Tajfel & Turner, 1979), possibly leading to less heritage cultural identity exploration. The effectiveness of these strategies depends on the inclusivity of the national identity, as well as characteristics such as phenotype and religious affiliation (Jugert et al., 2020). Due to the limited definition of what constitutes “being German”, i.e., being white, Christian-secular, and not of immigrant descent, the national identity is not accessible for all adolescents in Germany (Foroutan et al., 2014). Therefore, it would be important for future research to look into subgroups of adolescents of immigrant descent and consider experiences such as foreigner objectification in order to understand the varying effects. Adolescents could also lack opportunities to explore their heritage cultural identity in the German assimilationist context, which is an important pre-requisite for being able to engage in exploration (Verhoeven et al., 2019) and may also lead to lower heritage cultural identity exploration. This may be especially pronounced in smaller cities like Halle (Saale) compared to larger and more culturally diverse cities like Berlin.

### Basic Need Satisfaction and the Identity Project

Adolescents’ experiences of autonomy and relatedness across the three time points altered the effects of the Identity Project intervention on heritage cultural identity trajectories (research question 3). Yet, for autonomy support by teachers the pattern of findings was opposite than expected, with adolescents going through the Identity Project being more likely (and those not going through the Identity Project being less likely) to be in the low exploration trajectory when experiencing autonomy support by their teachers. One possible interpretation of this unexpected finding is that autonomy support by teachers, when combined with the incentives provided by the intervention to explore one’s heritage culture, may inadvertently create cognitive dissonance in adolescents. This cognitive dissonance could stem from the perception that autonomy support, intended to empower adolescents, may be associated with national culture values and thereby inadvertently convey assimilative messages, encouraging adolescents to align with the broader cultural norms within the German school environment (Coşkan et al., 2016). The simultaneous existence of both autonomy support and the incentive to explore their heritage culture could result in conflicting messages (Fuligni & Tsai, 2015), potentially leading to reduced exploration. Additional studies and in-depth qualitative research are needed to help shed more light on the underlying mechanisms involved in this phenomenon.

In regards to adolescents’ experiences of relatedness in the school context, peer belonging moderated the effect of the Identity Project on heritage cultural identity exploration, while relatedness support by teachers played a role in moderating the effect of the intervention on heritage cultural identity exploration, resolution and affirmation. Adolescents with stronger feelings of belonging to their peers, and who report better relationships with their teachers (perceived autonomy support) were more likely to be in higher exploration trajectories when participating in the Identity Project. Moreover, only when coupled with perceived relatedness support by teachers, were adolescents participating in the Identity Project intervention more likely to be in high resolution and affirmation trajectories. This further emphasizes the importance of differentiating different facets of relatedness that adolescents experience within the school environment when examining how need satisfaction plays a role in the relationship between interventions such as the Identity Project and heritage cultural identity development. In addition, the findings underscore that, despite the limited attention paid to affirmation in studies on the Identity Project, the intervention in conjunction with increased teacher support has the potential to contribute to adolescents having a more positive view of their heritage cultural identity.

These findings are in accordance with expectations that support of relatedness in the school context is an important condition for heritage cultural identity development processes promoted by the Identity Project. Forming meaningful connections with peers and teachers and fostering a sense of belonging and psychological safety (Vansteenkiste & Soenens, 2023), is essential for the intervention to unfold as intended. Moreover, it is intriguing that contrary to the study’s initial assumptions based on theory (Ryan & Deci, 2017) and previous studies (Luyckx et al., 2017), it appears that autonomy does not shape the effect of the Identity Project on heritage cultural identity development. Especially positive teacher-student relationships emerge as an important condition for intervention effects, indicating that a positive teacher-student relationship serves as fertile ground for the Identity Project and for achieving the desired intervention effects on heritage cultural identity development (Walton & Yeager, 2020). When going through the Identity Project, adolescents delve into personal narratives, engage with sensitive content, and may revisit past experiences of discrimination or feelings of alienation. In this context, teacher-student relationships emerge to be a crucial condition for adolescents to feel secure, encouraged, and empowered while navigating the intervention materials and activities, facilitating the desired positive outcomes of the Identity Project. The findings further suggest, that having teachers implement the Identity Project may enhance optimal conditions for the intervention. Studies have found that when teachers implement the Identity Project curriculum, this strengthens their relationships with their students (Umaña-Taylor, 2023), which may ultimately enhance the efficacy of the intervention.

Opposing patterns emerge for adolescents in the control condition, who did not go through the Identity Project. For those adolescents, the more relatedness support by teachers and the more peer belonging they reported, the more likely they were to be in lower exploration trajectories. These findings suggest that the intervention plays a pivotal role in redefining the dynamics between relatedness satisfaction and heritage cultural identity exploration among adolescents. The absence of the intervention’s structured support and encouragement for heritage cultural identity exploration may lead adolescents to interpret increased relatedness support of teachers as a signal to conform or adhere to established norms, again inadvertently communicating messages of assimilationism, a prominent feature of the German school context (Gries et al., 2021). Adolescents may further prioritize peer affiliations over independent exploratory behavior, particularly if those affiliations are perceived as socially normative or rewarding in the absence of clear exploration incentives. Research has shown that to avoid cognitive conflict, adolescents may choose certain identities prematurely if they align with the values and expectations of their close relationships (Côté & Levine, 2014). Therefore, satisfaction of the need for relatedness in the school context should be accompanied by opportunities to engage with heritage cultural identity, as to prevent meaningful relationships leading to adolescents feeling pressured to conform to certain norms in the school context and to encourage heritage cultural identity exploration.

While intervention effects on heritage cultural identity exploration and affirmation did not differ by immigrant descent, the intervention had a stronger positive effect on heritage cultural identity resolution for adolescents of non-immigrant descent, than for adolescents of immigrant descent. These findings reflect that adolescents of immigrant descent explore their heritage culture more, have a clearer sense of what their heritage culture means to them, and more positive feelings towards their heritage culture than adolescents of non-immigrant descent without the intervention, potentially leaving less room for further development facilitated by the Identity Project. The concept of culture is racialized in the German context, and often perceived as exclusively relevant for non-white, non-Christian-secular, immigrant individuals (Moffitt & Juang, 2019). The intervention is designed to increase exploration and resolution of heritage cultural identity for all adolescents by having them engage with their heritage cultural identity. By showing adolescents that culture is not something that is relevant only for adolescents of immigrant descent it may especially increase heritage culture identity resolution for adolescents of non-immigrant descent, helping to close the salience gap in heritage cultural identity resolution for adolescents of immigrant descent and non-immigrant descent. This nuanced finding underscores the importance of recognizing different entry points and strategies for various groups of adolescents within an intervention context (Ceccon et al., 2024). This is in line with other studies noting that some adolescents, particularly adolescents from majoritized groups, may require additional time to process and apply lessons learned from interventions like the Identity Project (Sladek et al., 2021), and may benefit from extended follow-up and support. By acknowledging the unique pathways of heritage cultural identity resolution for both adolescents of immigrant and non-immigrant descent, educators can implement tailored strategies that address their specific needs and challenges, ultimately fostering a more comprehensive approach in interventions to promote heritage cultural identity development.

### Limitations and Future Research

While the current study provides valuable insights into the interplay between autonomy and relatedness satisfaction in the school context and heritage cultural identity development, several limitations should be considered. The relatively small sample size and the emergence of small clusters can be challenging to interpret and may limit the generalizability of findings to broader populations. However, the small sample and cluster size of the current study (1) is consistent with other studies using similar methods (e.g., Juang et al., 2024), (2) acknowledges the challenges of conducting intervention studies in schools (e.g., Masia Warner & Fox, 2012), and (3) withstands sensitivity analyses, suggesting the findings are robust in regards to multivariate outliers. Nevertheless, future research with larger samples could provide a more comprehensive understanding of the relationships between autonomy and relatedness satisfaction and heritage cultural identity development and increase the validity of our findings. Moreover, the analyses were of exploratory nature and are a good starting point for further confirmatory research aiming to confirm the stability of these trajectories over time with larger samples, and their relationship with basic need satisfaction.

One reason for the relatively small sample size was the high amount of missing data, caused in large parts by disruptions due to the COVID-19 pandemic. Additionally, the measures utilized in this study were part of a lengthy questionnaire, contributing to the data collection challenges. The necessity of remote learning and the limitations it imposed on student engagement and data collection likely contributed to these drop-out rates. Participants without missing data were selected for the analyses, and these participants happened to be slightly younger than those with missing data. This could possibly be explained as adolescents who are older than the class average, for example due to having repeated a school year, are more likely to be absent at school (Gubbels et al., 2019). While this age difference was relatively small, it should be taken into account when considering the study’s findings. Future research could consider adapting data collection methods to better accommodate such disruptions and prevent the necessity of removing participants from analysis, such as more flexible data collection strategies that align with changing circumstances or shorter questionnaires. Additionally, establishing stronger collaborations with schools and educators could help ensure more consistent participation and completion of questionnaires (Horsfall et al., 2021), providing a more comprehensive and accurate representation of the target population.

The COVID-19 pandemic influenced the study beyond data collection issues. As the study took place in the school year 2021/22, adolescents’ everyday life was being impacted by the pandemic. Adolescents experienced a myriad of stressors, ranging from disruptions in daily routines and isolation from peers to heightened anxiety about the health and safety of their families, influencing their overall well-being (Ravens-Sieberer et al., 2022). This may have impacted their engagement with identity development processes in general (Fioretti et al., 2020), and the school-based Identity Project intervention specifically. This broader socio-emotional context within which the study took place should be taken into account when interpreting the study’s findings.

Another limitation of this study is that the measure of perceived teacher support of adolescents’ relatedness showed only limited (rather than good) reliability. Future research should thus use the long version of the Teacher as Social Context Questionnaire (Belmont et al., 1992) to test which items work best in the German context or use an alternative measure with better reliability in this context.

Lastly, the specific cultural and educational context – German schools with a substantial proportion of adolescents from immigrant backgrounds – has to be considered when interpreting the findings. It is important to note that while there are known cultural variations in the concept of autonomy, particularly concerning separation from parents (Kağıtçıbaşı, 2013), the study focused on autonomy as it relates to intrinsic motivation and autonomy-supportive teaching practices. The theoretical foundations of the study, including Self-Determination Theory (Ryan & Deci, 2017), Erikson’s identity framework (1968), and stage-environment fit theory (Eccles & Roeser, 2011), suggest that while the findings were derived from a specific context, the principles of identity development, and autonomy and relatedness satisfaction likely hold universal relevance for adolescents across diverse societies. These theoretical and empirical frameworks emphasize the universal quest for identity, the importance of basic psychological needs, and the influence of supportive environments, implying that the findings have broader implications beyond the immediate context of the current study (Vansteenkiste et al., 2020). Nonetheless, replicating this study in different cultural and educational contexts could help further clarify how variations in autonomy and relatedness, as well as the balance between autonomy and relatedness (see Coşkan et al., 2016), contribute to adolescent development across cultures.

Moreover, in line with other studies in the European context and due to the socio-cultural variations in understanding ethnicity, race, and culture, including a taboo around discussing race, ethnic-racial identity was conceptualized as heritage cultural identity in the current study (Juang et al., 2021). It is essential to recognize that while heritage cultural identity overlaps with ethnic-racial identity, and culture is racialized in the German context, this measure does not explicitly address racialized identities. Thus, while heritage cultural identity does function similarly to racialized identities in the United States, future research should aim to explicitly measure racialized identities to provide a comprehensive understanding of identity development in contexts like Germany, where race remains salient but is not explicitly discussed.

## Conclusion

As heritage cultural identity development is an important task in adolescence, it is crucial to understand the contextual factors that promote this development in the school context, particularly within school-based interventions. Autonomy and relatedness, without incentives to engage in heritage cultural identity development such as offered by the Identity Project intervention, produce varying effects on heritage cultural identity development. However, teacher-student relationships emerge as an important and necessary condition for the intervention to promote positive heritage cultural identity development in adolescents. Furthermore, the study reveals differing paths of heritage cultural identity development for adolescents of immigrant and non-immigrant descent; heritage cultural identity is more salient for adolescents of immigrant descent before the intervention, while adolescents of non-immigrant descent may benefit more from interventions such as the Identity Project. These findings hold important implications for refining the design and implementation of school-based interventions aimed at promoting adolescents’ heritage cultural identity, emphasizing that in order to effectively promote heritage cultural identity development in adolescents, not only are effective and tailored interventions needed, but also support from the broader context, specifically strong teacher-student relationships.

## Supplementary information

Supplementary Information

## Footnotes

1 In studies conducted in the United States, the term “ethnic-racial identity” is commonly used, whereas in Europe, particularly in studies focusing on heritage cultural identity, the term “heritage cultural identity” is utilized. Similarly, when describing minoritized students in the United States, the term “ethnic-racial minoritized” is employed, whereas in Europe, individuals who are perceived as immigrants based on phenotype, culture, or religion are referred to as being of “immigrant descent”. For further elaboration, please refer to the section “Ethnic-Racial Identity Development in the German Context.”

2 Self-Determination Theory posits that next to autonomy and relatedness, competence is a basic need, referring to feeling capable and effective in various endeavors. While competence is undoubtedly an important aspect of individuals’ development, and all three needs have to be satisfied in order to have good psychological health and personal well-being (Ryan & Deci, 2017), due to extensive research emphasizing the unique and critical roles autonomy and relatedness play in adolescents’ identity development (Soenens & Vansteenkiste, 2023), the current study prioritizes these needs over competence.

3 These findings should be interpreted in the context of latent profile cluster analyses, i.e. analyses looking at the trajectories of sub-groups of the sample. The findings do not preclude the possibility that overall, the intervention increases average-level heritage cultural identity scores of students. The findings only indicate, that what identity trajectory adolescents belong to, is not solely predicted by whether they are in the intervention or control condition.
