# Supplementary material for: Promoting Adolescents’ Heritage Cultural Identity Development: Exploring the Role of Autonomy and Relatedness Satisfaction in School-Based Interventions
Source: J Youth Adolesc. 2024 May 24;53(11):2460–79. doi: 10.1007/s10964-024-02017-3 (PMC11467014; doi:10.1007/s10964-024-02017-3)
Supplement: Supplementary file 1 — Supplementary Information [file 10964_2024_2017_MOESM1_ESM.pdf]

Promoting Adolescents' Heritage Cultural Identity Development in the Context of the Identity Project in Germany: The Role of Autonomy and Relatedness Basic Needs Satisfaction

Online Supplementary Material

November 17th, 2023

Content

Table S1. Descriptives exploration clusters. 2

Table S2. Descriptives resolution clusters. 3

Table S3. Descriptives affirmation clusters. 4

Table S4. Results of the best-fitting multinomial model for heritage cultural identity exploration. 5

Table S5. Result of the best-fitting multinomial model for heritage cultural identity resolution. 6

Table S6. Result of the best-fitting linear model for heritage cultural identity affirmation. 7

Figure S1. Sensitivity analysis heritage cultural identity exploration. 8

Figure S2. Sensitivity analysis heritage cultural identity resolution. 9

Figure S3. Sensitivity analysis heritage cultural identity affirmation. 10

Figure S4. Heritage cultural identity exploration – main effect of immigrant descent. 11

Figure S5. Heritage cultural identity exploration – main effect of intrinsic motivation. 12

Figure S6. Heritage cultural identity exploration – interaction intervention X perceived autonomy support by teachers. 13

Figure S7. Heritage cultural identity exploration – interaction intervention X perceived relatedness support by teachers. 14

Figure S8. Heritage cultural identity exploration – interaction intervention X peer belonging. 15

Figure S9. Heritage cultural identity resolution – interaction intervention X immigrant descent. 16

Figure S10. Heritage cultural identity resolution – interaction intervention X perceived relatedness support by teachers. 17

Figure S11. Heritage cultural identity affirmation – main effect of immigrant descent. 18

Figure S12. Heritage cultural identity affirmation – main effect of peer belonging. 19

Figure S13. Heritage cultural identity affirmation – interaction intervention X perceived relatedness support by teachers. 20

Supplementary References: *R* packages. 21

Table S1. Descriptives exploration clusters.

	**Cluster 1: low stable exploration (*n* = 19)**	**Cluster 2: medium stable exploration (*n* = 107)**	**Cluster 3: high stable exploration (*n* = 72)**
**Immigrant descent**			
Adolescents of Immigrant descent *n* = 81 (40.91%)	*n* = 8 (42.11%)	*n* = 26 (24.30%)	*n* = 47 (65.28%)
Adolescents of Non-immigrant descent *n* = 117 (59.09%)	*n* = 11 (57.89%)	*n* = 81 (75.70%)	*n* = 25 (34.72%)
**Intervention group**			
Intervention *n* = 97 (48.99%)	*n* = 10 (52.63%)	*n* = 51 (47.66%)	*n* = 36 (50.00%)
Control *n* = 101 (51.01%)	*n* = 9 (47.37%)	*n* = 56 (52.34%)	*n* = 36 (50.00%)

Note. There is a difference of immigrant descent in the three clusters (X^2 (2, N = 198) = 29.91, p < .001, V = .39). There is no evidence of difference of intervention in the three clusters (X^2 (2, N = 198) = 0.21, p = 0.902, V = .03).

Table S2. Descriptives resolution clusters.

	**Cluster 1: decrease T2-increase T3 resolution n = 12**	**Cluster 2: medium stable resolution n = 77**	**Cluster 3: low decrease resolution n = 35**	**Cluster 4: high increase resolution n = 74**
**Immigrant descent**				
Adolescents of Immigrant descent n = 81 (40.91%)	n = 3 (25.00%)	n = 25 (32.47%)	n = 3 (8.57%)	n = 50 (67.57%)
Adolescents of Non-immigrant descent n = 117 (59.09%)	n = 9 (75.00%)	n = 52 (67.53%)	n = 32 (91.43%)	n = 24 (32.43%)
**Intervention group**				
Intervention n = 97 (48.99%)	n = 5 (41.67%)	n = 38 (49.35%)	n = 17 (48.57%)	n = 37 (50.00%)
Control n = 101 (51.01%)	n = 7 (58.33%)	n = 39 (50.65%)	n = 18 (51.43%)	n = 37 (50.00%)

Note. There is a difference of immigrant descent in the four clusters (X^2 (3, N = 198) = 40.42, p < .001, V = .45). There is no evidence of difference of intervention in the four clusters (X^2 (3, N = 198) = 0.29, p = 0.961, V = .04).

Table S3. Descriptives affirmation clusters.

	**Cluster 1: medium affirmation (*n* = 159)**	**Cluster 2: high affirmation (*n* = 39)**
**Immigrant descent**		
Adolescents of Immigrant descent *n* = 81 (40.91%)	*n* = 47 (29.56%)	*n* = 34 (87.18%)
Adolescents of Non-immigrant descent *n* = 117 (59.09%)	*n* = 112 (70.44%)	*n* = 5 (12.82%)
**Intervention group**		
Intervention *n* = 97 (48.99%)	*n* = 81 (50.94%)	*n* = 16 (41.03%)
Control *n* = 101 (51.01%)	*n* = 78 (49.06%)	*n* = 23 (58.97%)

Note. There is a difference of immigrant descent in the two clusters ($X^2(1, N = 198) = 43.01$, $p < .001$, $V = .47$). There is no difference of intervention group in the two clusters ($X^2(1, N = 198) = 1.23$, $p = 0.267$, $V = .08$).

Table S4. Results of the best-fitting multinomial model for heritage cultural identity exploration.

		Cluster 1: low stable		Cluster 3: high stable	
	Omnibus X^2*(df)*	*β(SE)*	*Odds Ratio*	*β(SE)*	*Odds Ratio*
Independent variable					
Gender	5.54(2)	-1.13(.62)	0.32	0.30(.35)	1.35
Intervention	0.09(2)	0.10(.64)	1.10	-0.07(.35)	0.93
Immigrant descent	30.10(2)***	1.48(.61)*	4.41	1.82(.36)***	6.17
Perceived autonomy support by teachers	0.80(2)	-0.71(.52)	0.49	0.05(.32)	1.05
Perceived relatedness support by teachers	1.09(2)	0.43(.47)	1.53	-0.26(.29)	0.77
Peer belonging	0.17(2)	0.81(.48)	2.24	0.36(.25)	1.43
Intrinsic motivation	12.04(2)**	-1.10(.35)**	0.33	0.01(.20)	1.01
Intervention X Perceived autonomy support by teachers	8.94(2)*	1.97(.72)**	7.15	-0.07(.46)	0.93
Intervention X Perceived relatedness support by teachers	11.51(2)**	-0.97(.68)	0.38	1.22(.49)*	3.39
Intervention X Peer belonging	9.11(2)*	-1.64(.65)*	0.19	-.073(.40)	0.48

Note. Nagelkerke's R^2 = .36. For the dependent variable, the reference category is cluster 2: medium stable cluster. As for the independent variables, the baseline category for gender was male, for intervention was the control group, and for immigrant descent was non-immigrant descent. **p* <.05, ***p*< 0.01, ****p*<.001.

Table S5. Result of the best-fitting multinomial model for heritage cultural identity resolution.

		Cluster 1: decrease T2 – increase T3		Cluster 3: low decrease		Cluster 4: high increase	
	Omnibus X^2*(df)*	*β(SE)*	*Odds Ratio*	*β(SE)*	*Odds Ratio*	*β(SE)*	*Odds Ratio*
Independent variable							
Intervention	0.17(3)	-0.85(0.86)	0.43	-0.21(0.48)	0.81	0.67(0.54)	1.96
Immigrant descent	45.18(3)***	-0.81(1.17)	0.44	-15.72(0.38)***	0.00	2.33(0.56)***	10.31
Perceived relatedness support by teachers	13.18(3)**	-0.49(0.39)	0.61	-0.42(0.28)	0.66	-0.24(0.27)	0.78
Peer belonging	7.57(3)	0.67(0.39)	1.96	-0.05(0.22)	0.95	0.39(0.19)*	1.48
Intervention X Immigrant descent	9.50(3)*	0.85(1.54)	2.35	14.47(0.38)***	1930864	-1.56(0.74)*	0.21
Intervention X Perceived relatedness support by teachers	7.98(3)*	-0.70(0.64)	0.50	-0.30(0.44)	0.74	0.96(0.45)*	2.62

Note. Nagelkerke's R^2 = .38. For the dependent variable, the reference category is cluster 2: medium stable cluster. As for the independent variables, the baseline category for intervention was the control group and for immigrant descent was non-immigrant descent. **p* <.05, ***p*< 0.01, ****p*<.001.

Table S6. Result of the best-fitting linear model for heritage cultural identity affirmation.

		Cluster 2: high affirmation	
	Omnibus X^2*(df)*	*β(SE)*	*Odds Ratio*
Independent variable			
Age	2.97(1)	-0.50(0.30)	0.61
Intervention	2.27(1)	-1.05(0.53)*	0.35
Immigrant descent	50.83(1)***	3.29(0.57)***	26.94
Perceived relatedness support by teachers	5.18(1)*	0.17(0.29)	1.19
Peer belonging	4.23(1)*	0.49(0.24)*	1.63
Intervention X Perceived relatedness support by teachers	6.14(1)*	1.48(0.64)*	4.37

Note. Nagelkerke's R^2 = .57. A multinomial model did not converge. For the dependent variable, the reference category is cluster 1: medium affirmation cluster. As for the independent variables, the baseline category for intervention was the control group and for immigrant descent was non-immigrant descent. *p <.05, **p< 0.01, ***p<.001.

Figure S1. Sensitivity analysis heritage cultural identity exploration.

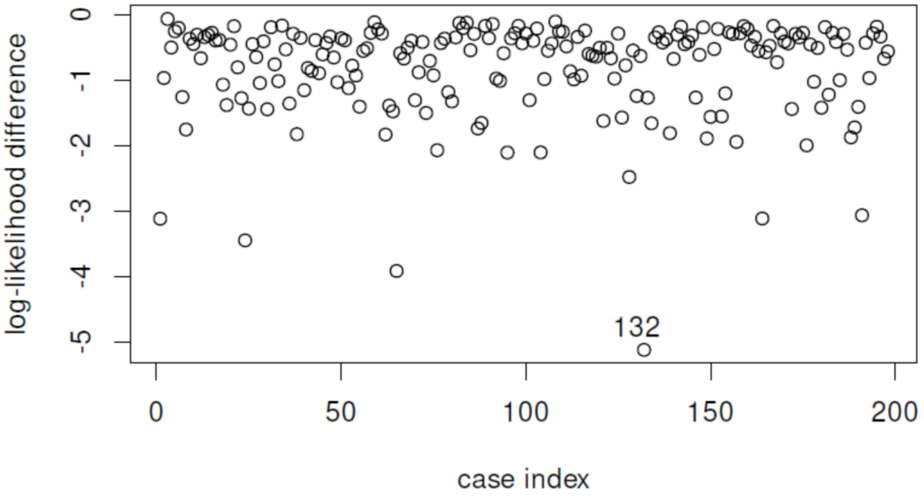

Note. Sensitivity analyses for the best multinomial model predicting heritage cultural identity exploration (n = 198). Log-likelihood difference is the difference between the log-likelihood of model fitted on the whole sample and the log-likelihood of the model fitted to the sample without a considered case. The index of the most influential case, number 132, is presented. However: 1) the signs of the parameters in the full model coincide with those in the model without case 132; 2) only one estimated effect, namely gender, differs in terms of statistical significance (at the 0.05 level). Specifically, while in the full model, gender is not statistically significant ($X^2(2, N = 198) = 5.54$, $p = 0.063$), in the model without case 132, gender is statistically significant ($X^2(2, N = 198) = 12.25$, $p = 0.002$). Considering the overall minimal differences between the findings of the full model and the model without case 132 and following a conservative perspective we have decided not to exclude this case.

Figure S2. Sensitivity analysis heritage cultural identity resolution.

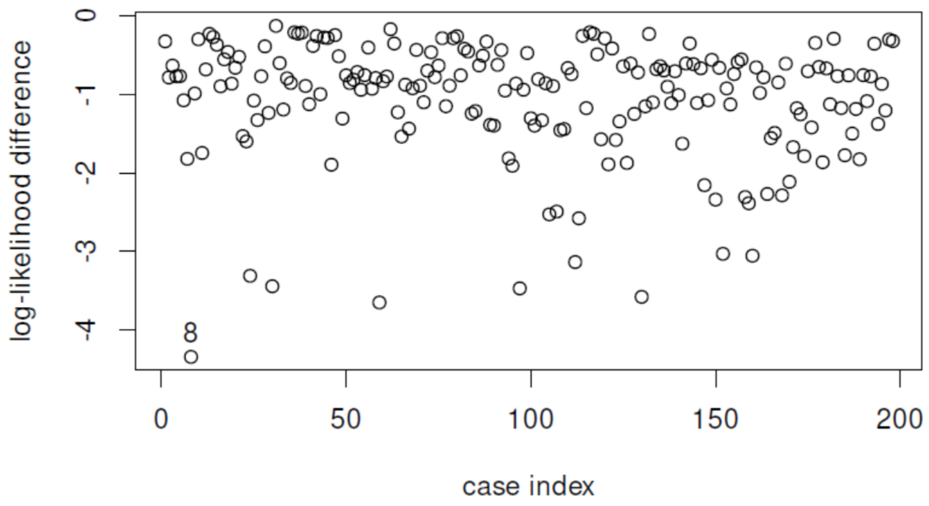

Note. Sensitivity analyses for the best multinomial model predicting heritage cultural identity resolution (n = 198). Log-likelihood difference is the difference between the log-likelihood of model fitted on the whole sample and the log-likelihood of the model fitted to the sample without a considered case. The index of the most influential case, number 8, is presented. However: 1) the signs of the parameters in the full model coincide with those in the model without case 8; 2) only one estimated effect, namely peer belonging, differs in terms of statistical significance (at the 0.05 level). Specifically, while in the full model, peer belonging is not statistically significant ($X^2(3, N = 198) = 7.57$, $p = 0.056$), in the model without case 8, peer belonging is statistically significant ($X^2(3, N = 198) = 14.25$, $p = 0.003$). Considering the overall minimal differences between the findings of the full model and the model without case 8 and following a conservative perspective we have decided not to exclude this case.

Figure S3. Sensitivity analysis heritage cultural identity affirmation.

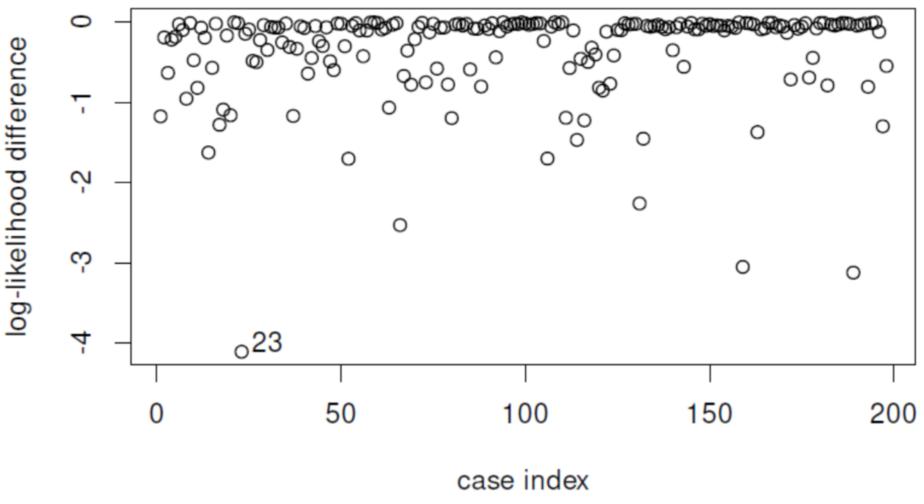

Note. Sensitivity analyses for the best multinomial model predicting heritage cultural identity affirmation (*n* = 198). Log-likelihood difference is the difference between the log-likelihood of model fitted on the whole sample and the log-likelihood of the model fitted to the sample without a considered case. The index of the most influential case, number 23, is presented. However: 1) the signs of the parameters in the full model coincide with those in the model without case 23; 2) all estimated effects did not differ in terms of statistical significance (at the .05 level) between the full model and the model without case 23. Based on this results, we have decided not to exclude the case 23.

Figure S4. Heritage cultural identity exploration – main effect of immigrant descent.

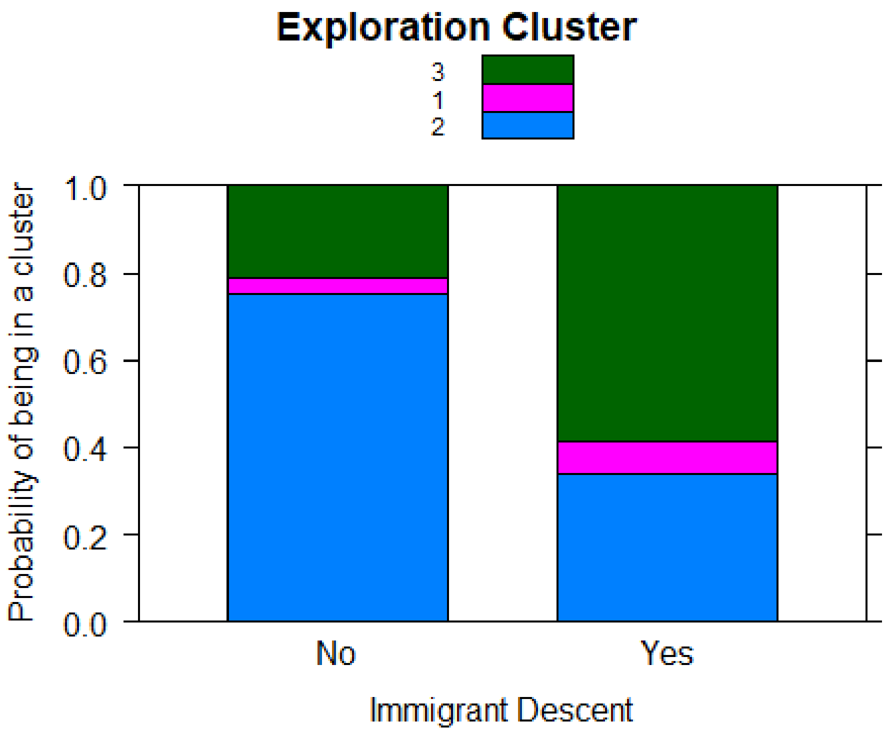

Note. Cluster 1 represents the low stable, cluster 2 the medium stable, and cluster 3 the high stable exploration cluster. There is a main effect of immigrant descent. Students of immigrant descent have an increased probability of being in the low stable exploration cluster [1] and in the high stable exploration cluster [3].

Figure S5. Heritage cultural identity exploration – main effect of intrinsic motivation.

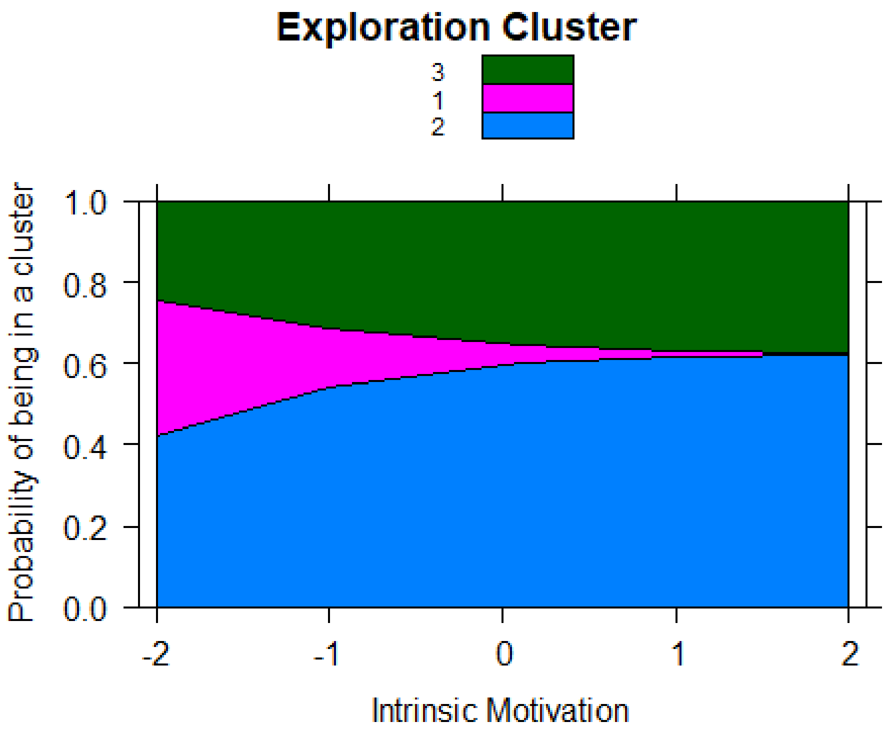

Note. Cluster 1 represents the low stable, cluster 2 the medium stable, and cluster 3 the high stable exploration cluster. There is a main effect of intrinsic motivation when comparing the low-stable and medium-stable exploration clusters. As the students' intrinsic motivation increases, the probability for being in the low-stable exploration cluster [1] decreases.

Figure S6. Heritage cultural identity exploration – interaction intervention X perceived autonomy support by teachers.

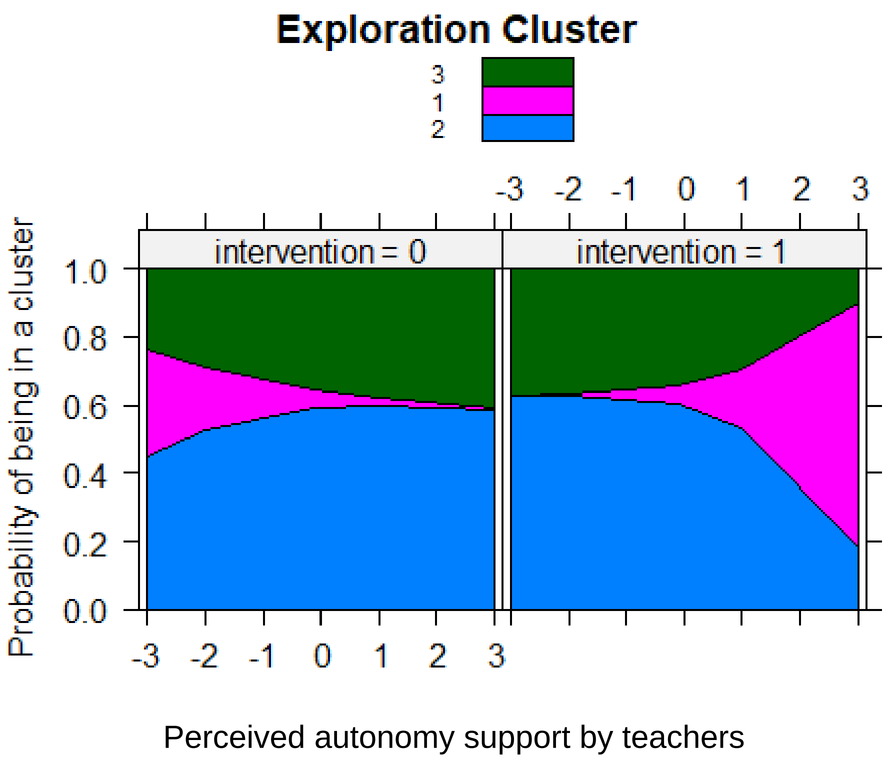

Note. Cluster 1 represents the low stable, cluster 2 the medium stable, and cluster 3 the high stable exploration cluster. There is an interaction between intervention condition and perceived autonomy support by teachers when comparing the low-stable and medium-stable exploration clusters. In the control condition, as the students' perceived autonomy support by teachers increases, the probability for being in the low-stable exploration cluster [1] decreases. However, in the intervention condition, as the students' perceived autonomy support by teachers increases, the probability for being in the low-stable exploration cluster [1] increases.

Figure S7. Heritage cultural identity exploration – interaction intervention X perceived relatedness support by teachers.

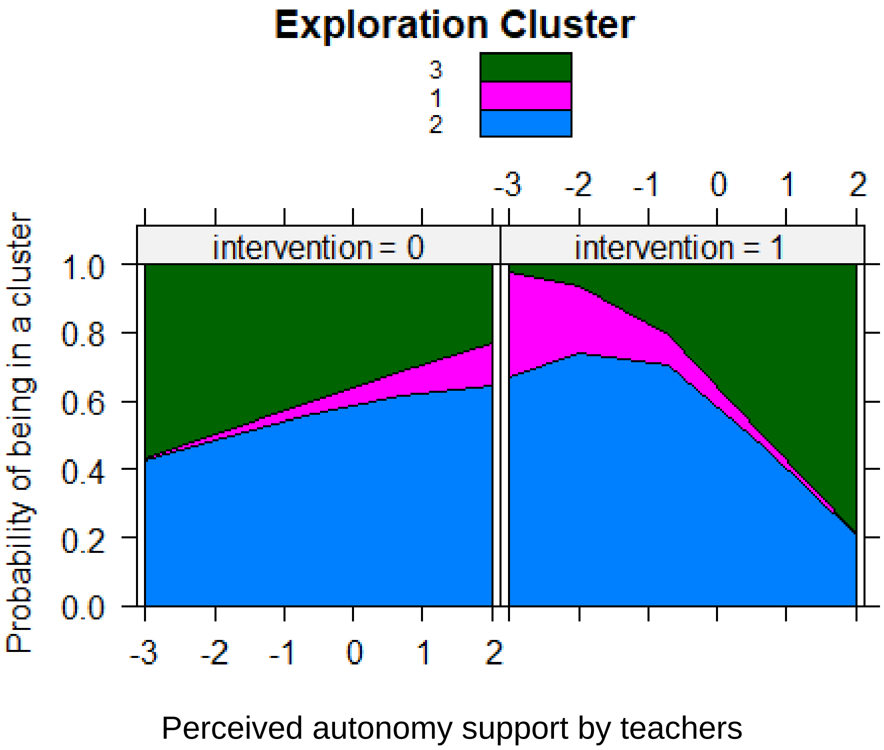

Note. Cluster 1 represents the low stable, cluster 2 the medium stable, and cluster 3 the high stable exploration cluster. There is an interaction between intervention condition and perceived relatedness support by teachers when comparing the high-stable and medium-stable exploration clusters. In the control condition, as the students' perceived relatedness support by teachers increases, the probability for being in the high-stable exploration cluster [3] decreases. However, in the intervention condition, as the students' perceived relatedness support by teachers increases, the probability for being in the high-stable exploration cluster [3] increases.

Figure S8. Heritage cultural identity exploration – interaction intervention X peer belonging.

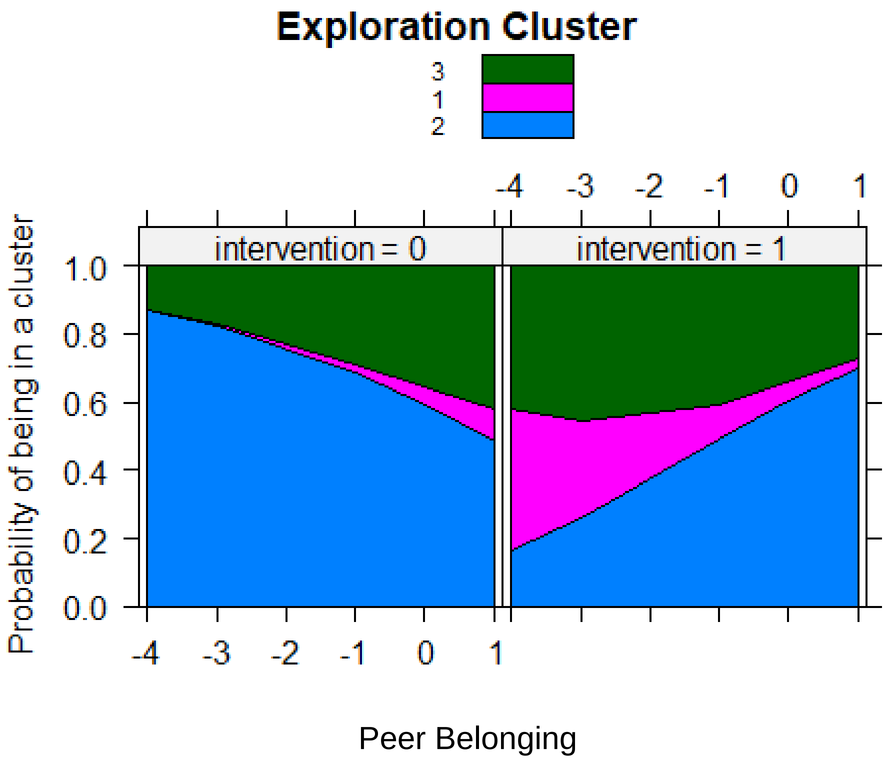

Note. Cluster 1 represents the low stable, cluster 2 the medium stable, and cluster 3 the high stable exploration cluster. There is an interaction between intervention condition and peer belonging when comparing the low-stable and medium-stable exploration clusters. In the intervention condition, as the students' feeling of peer belonging increase, the probability for being in the low-stable exploration cluster [1] decreases. However, in the control condition, as the students' peer belonging increases, the probability for being in the low-stable exploration cluster [1] also increases.

Figure S9. Heritage cultural identity resolution – interaction intervention X immigrant descent.

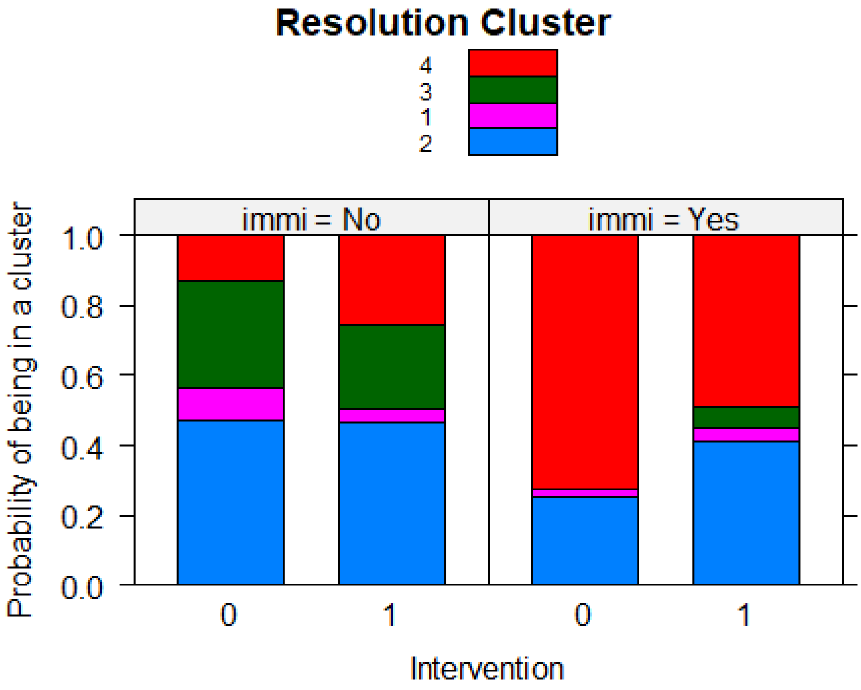

Note. Cluster 1 represents the decrease T2 – increase T3, cluster 2 the medium stable, cluster 3 the low decrease, and cluster 4 the high increase resolution cluster. There is an interaction between the intervention group and students' status of immigrant descent, when comparing the medium-stable and the low-decrease resolution cluster, and the medium-stable and the high-increase resolution cluster. Students of immigrant descent, are less likely to be in low-decrease resolution cluster [3] and more likely to be in the high-increase resolution cluster [4] than students of non-immigrant descent. This is more pronounced in the control condition, where there are no students of immigrant descent in the low-decrease resolution cluster [3], and the highest probability of students being in the high-increase resolution cluster [4]. Furthermore, for students of non-immigrant descent, the probability of being in the high-increase resolution cluster [4] increases when in the intervention group, and being in the low-decrease resolution cluster [3] decreases when in the intervention group.

Figure S10. Heritage cultural identity resolution – interaction intervention X perceived relatedness support by teachers.

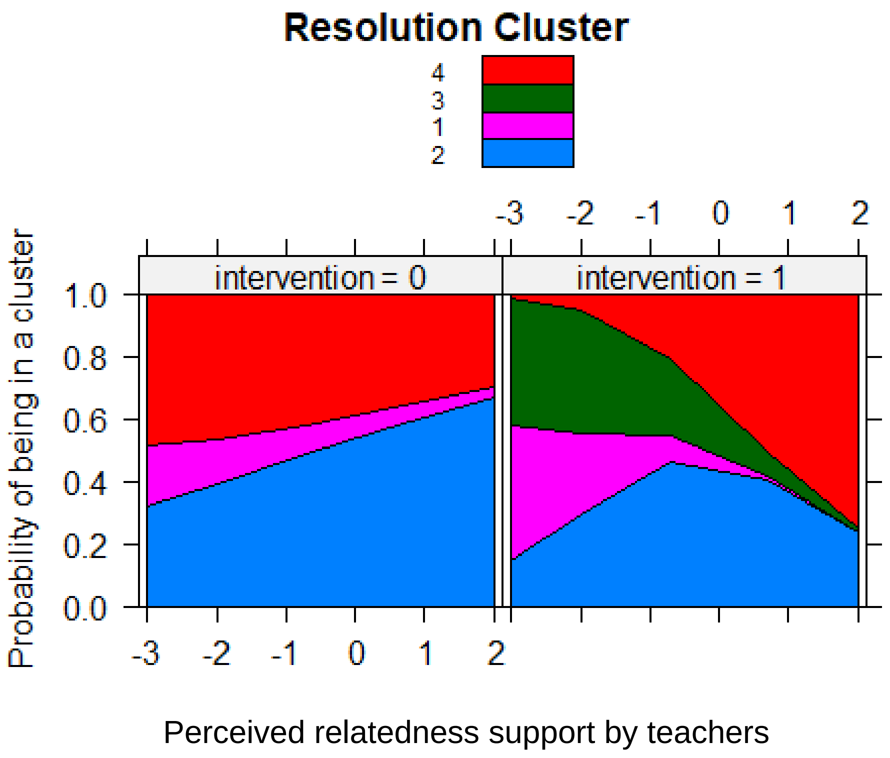

Note. Cluster 1 represents the decrease T2 – increase T3, cluster 2 the medium stable, cluster 3 the low decrease, and cluster 4 the high increase resolution cluster. There is an interaction between the intervention group and perceived relatedness support by teachers when comparing the medium-stable and high increase resolution cluster. In the intervention condition, an increase in perceived relatedness support by teachers increases the probability of being in the high increase resolution cluster [4]. This is not the case in the control condition.

Figure S11. Heritage cultural identity affirmation – main effect of immigrant descent.

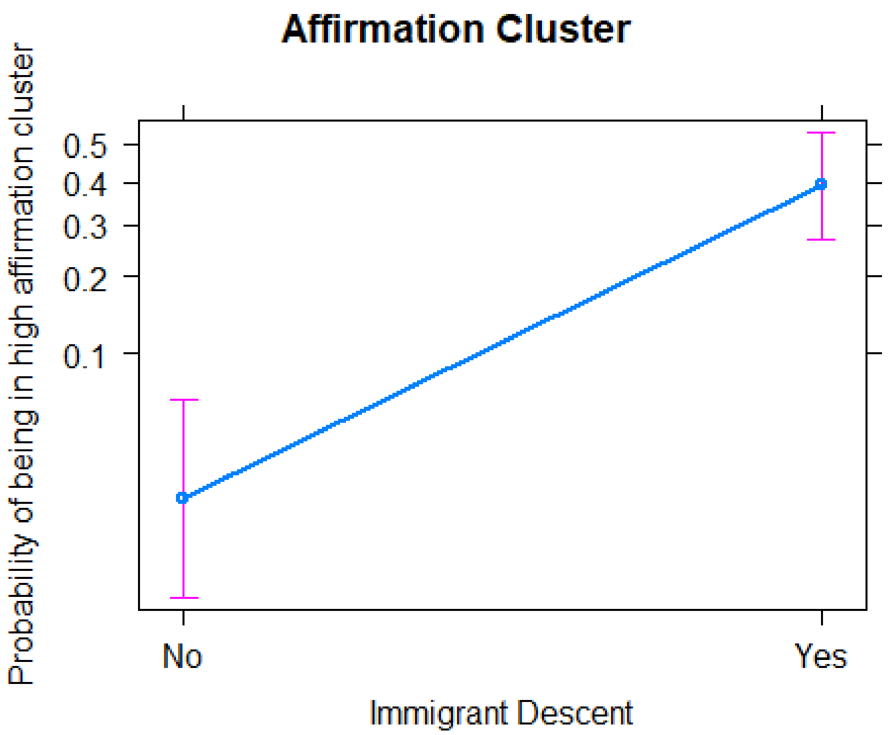

Note. The Y-axis depicts the probability of being in the high affirmation cluster, compared to the medium affirmation cluster. There is a main effect of immigrant descent. There is a higher probability of being in the high affirmation cluster for students of immigrant descent, compared to students of non-immigrant descent.

Figure S12. Heritage cultural identity affirmation – main effect of peer belonging.

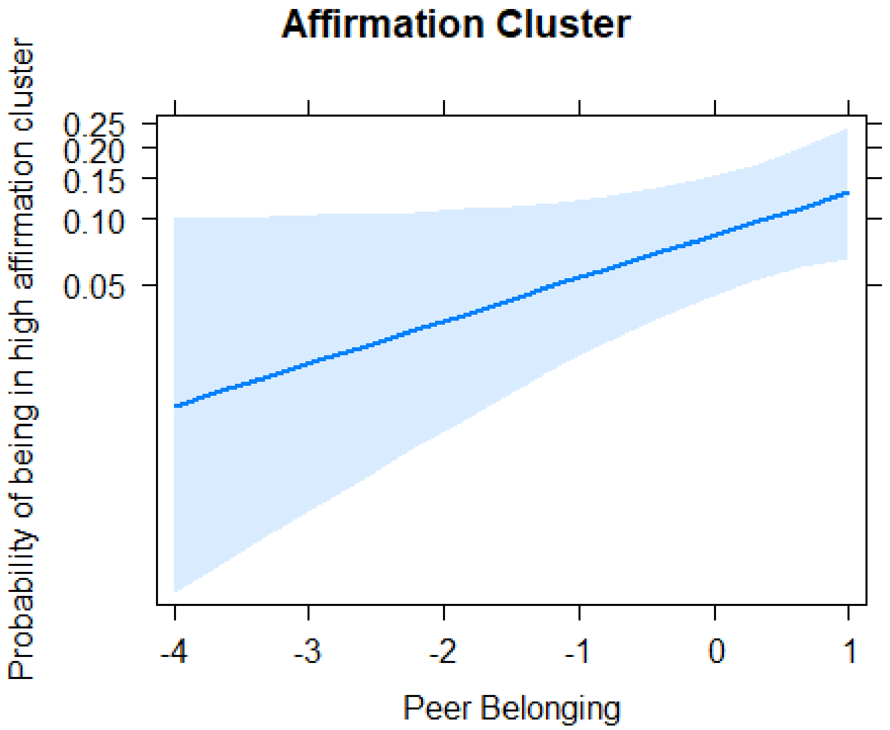

Note. The Y-axis depicts the probability of being in the high affirmation cluster, compared to the medium affirmation cluster. There is a main effect of peer belonging. Students with high levels of peer belonging are more likely to be in the high affirmation cluster

Figure S13. Heritage cultural identity affirmation – interaction intervention X perceived relatedness support by teachers.

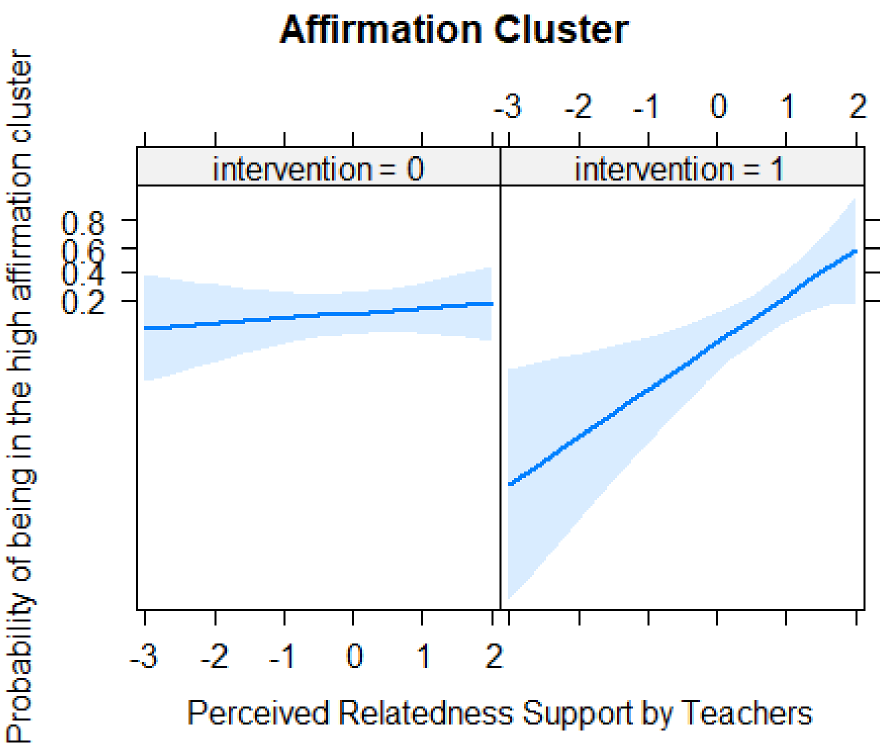

Note. The Y-axis depicts the probability of being in the high affirmation cluster, compared to the medium affirmation cluster. There is an interaction between intervention condition and perceived relatedness support by teachers. In the control condition, there is no difference in probability of being in the high affirmation cluster dependent on perceived relatedness support by teachers. In the intervention condition however, the probability of being in the high affirmation cluster is increased for students with higher levels of perceived relatedness support by teachers.

Supplementary References: *R* packages.

car package: Fox, J. & Weisberg, S. (2019). *An R Companion to Applied Regression* (3rd ed.). Sage. https://socialsciences.mcmaster.ca/jfox/Books/Companion/

mclust package: Scrucca, L., Fop, M., Murphy, T. B. & Raftery A. E. (2016). *mclust* 5: Clustering, classification and density estimation using Gaussian finite mixture models. *The R Journal, 8*(1), 289-317. https://doi.org/10.32614/RJ-2016-021

nnet package: Venables, W. N. & Ripley, B. D. (2002). *Modern Applied Statistics with S. Fourth Edition*. Springer. https://www.stats.ox.ac.uk/pub/MASS4/

stats package: R Core Team. (2022). R: *A language and environment for statistical computing*. Vienna, Austria: R Foundation for Statistical Computing.
